# Identifying a Hypoxia-Related Long Non-Coding RNAs Signature to Improve the Prediction of Prognosis and Immunotherapy Response in Hepatocellular Carcinoma

**DOI:** 10.3389/fgene.2021.785185

**Published:** 2021-11-30

**Authors:** Pingfei Tang, Weiming Qu, Taoli Wang, Minji Liu, Dajun Wu, Lin Tan, Hongbing Zhou

**Affiliations:** ^1^ Department of Digestive Diseases, Zhuzhou Central Hospital, The Affiliated Zhuzhou Hospital of Xiangya Medical College of Central South University, Zhuzhou, China; ^2^ Department of Pathology, Zhuzhou Central Hospital, The Affiliated Zhuzhou Hospital of Xiangya Medical College of Central South University, Zhuzhou, China

**Keywords:** hypoxia, lncRNA (long non-coding RNA), hepatocellular carcinoma, prognostic signature, tumor immune microenvironment, immunotherapy response

## Abstract

**Abstract Background:** Both hypoxia and long non-coding RNAs (lncRNAs) contribute to the tumor progression in hepatocellular carcinoma (HCC). We sought to establish a hypoxia-related lncRNA signature and explore its correlation with immunotherapy response in HCC.

**Materials and Methods:** Hypoxia-related differentially expressed lncRNAs (HRDELs) were identified by conducting the differential gene expression analyses in GSE155505 and The Cancer Genome Atlas (TCGA)- liver hepatocellular carcinoma (LIHC) datasets. The HRDELs landscape in patients with HCC in TCGA-LIHC was dissected by an unsupervised clustering method. Patients in the TCGA-LIHC cohort were stochastically split into the training and testing dataset. The prognostic signature was developed using LASSO (least absolute shrinkage and selection operator) penalty Cox and multivariable Cox analyses. The tumor immune microenvironment was delineated by the single-sample gene set enrichment analysis (ssGSEA) algorithm. The Tumor Immune Dysfunction and Exclusion (TIDE) algorithm was applied to evaluate the predictive value of the constructed signature in immunotherapeutic responsiveness.

**Results:** A total of 55 HRDELs were identified through integrated bioinformatical analyses in GSE155505 and TCGA-LIHC. Patients in the TCGA-LIHC cohort were categorized into three HRDELs-specific clusters associated with different clinical outcomes. The prognostic signature involving five hypoxia-related lncRNAs **(** *LINC00869*, *CAHM*, *RHPN1-AS1*, *MKLN1-AS*, and *DUXAP8*) was constructed in the training dataset and then validated in the testing dataset and entire TCGA-LIHC cohort. The 5-years AUC of the constructed signature for prognostic prediction reaches 0.705 and is superior to that of age, AJCC stage, and histopathological grade. Patients with high-risk scores consistently had poorer overall survival outcomes than those with low-risk scores irrespective of other clinical parameters status. The low-risk group had more abundance in activated CD8^+^ T cell and activated B cell and were predicted to be more responsive to immunotherapy and targeted therapy than the high-risk group.

**Conclusion:** We established a reliable hypoxia-related lncRNAs signature that could accurately predict the clinical outcomes of HCC patients and correlate with immunotherapy response and targeted drug sensitivity, providing new insights for immunotherapy and targeted therapy in HCC.

## Introduction

Liver malignancy is the sixth frequent malignant disease with a growth of 905,677 new cases in 2020 and becomes the third leading cause of tumor-associated death worldwide (Sung, et al., 2021). Hepatocellular carcinoma (HCC) occupies nearly 90% of patients with primary liver cancer (Forner, et al., 2018). Owing to lacking apparent symptoms in the initial stage, many cases were diagnosed in the advanced stage in HCC and lost the curative surgeon opportunity. Targeted therapy such as sorafenib represents the first-line strategy for advanced-stage cases. However, the overall clinical outcomes are still far from satisfactory owing to the emerged resistance of sorafenib (Zhu, et al., 2017). In recent years, immunotherapy based on immune checkpoint inhibitors has brought favorable treatment benefits in several solid tumors (Darvin, et al., 2018), including hepatocellular carcinoma (El-Khoueiry, et al., 2017). Nevertheless, only a subgroup of HCC patients responded to immunotherapy and most of them died of tumor recurrence and metastasis. It is of paramount importance to explore new prognostic biomarkers and potential predictors of immunotherapeutic response for HCC.

Hypoxia is a specific feature in solid tumors (Pouysségur, et al., 2006). Owing to the fast expansion of tumor cells and abnormal vascularization, the tumor microenvironment suffers from insufficient oxygen and nutrition. The hypoxia-inducible factor-1 alpha (HIF-1α) signaling plays a momentous role in the regulation of tumor development, metastasis, recurrence, and drug resistance in the hypoxic tumor microenvironment (LaGory and Giaccia, 2016; Rankin and Giaccia, 2016). HIF-1α can enhance the stemness of HCC cell lines in hypoxia exposure, and the knockdown of HIF1α in HCC cells can effectively downturn the extracellular acidification rate under hypoxic conditions (Ling, et al., 2020).

Evidence has suggested that lncRNAs are involved in the dysregulation of gene expression and signaling pathways closely linked to tumor initiation, progression, and distant metastasis (Slack and Chinnaiyan, 2019). Recently, many studies have revealed that lncRNAs also participate in the hypoxia-response process of cancer cells (Choudhry, et al., 2016; Huan, et al., 2020), and the interplay between hypoxia and lncRNAs is connected with tumor aggression and metastasis (Wang, et al., 2021). In HCC, hypoxia exposure promotes epithelial-to-mesenchymal transition (EMT) and distant metastasis of HCC cells with overexpression of lncRNA AGAP2-AS1, while the knockdown of AGAP2-AS1 can reverse the aggressive phenotype (Liu, et al., 2019). Thus, we speculate that hypoxia-related lncRNAs tightly affect the progression of HCC and have a substantial influence on the clinical outcomes of HCC patients. Moreover, the hypoxic tumor microenvironment can drive cancer cells to an immune resistance phenotype and contribute to the resistance to immunotherapy (Abou Khouzam, et al., 2020). To our knowledge, there is still a lack of hypoxia-related lncRNAs signature that can accurately predict the prognosis and immunotherapeutic responsiveness in HCC.

In the current study, we sought to microdissect the hypoxia-related lncRNAs landscape in HCC and establish a hypoxia-related lncRNAs prognostic signature in HCC patients in the TCGA-LIHC cohort. We also in-depth investigated the association of the prognostic signature with tumor immune infiltration pattern, targeted-drug sensitivity, and immunotherapy response. Our findings may improve the prognostic prediction and personalized treatment management of immunotherapy in HCC.

## Materials and Methods

### Data Preparation

The FPKM profiles of the transcriptome sequencing data of HCC patients in the TCGA -LIHC cohort were publicly obtained from TCGA database. We then transformed the FPKM values into the log_2_-transformed TPM (Transcripts Per Million) values for further analysis. The microarray dataset GSE155505 consisting of human HCC cells treated with hypoxia or normoxia was publicly obtained from Geo Expression Ombimus (GEO) database.

The TCGA-LIHC project comprises 374 primary HCC tumor samples and 50 normal specimens, and their clinical data were publicly obtained from the cBioPortal database (Cerami, et al., 2012). Patients were included in the present study based on the following criteria: 1) patients had the complete overall survival (OS) time and status; 2) patients with OS time <30 days were excluded for the reason that these patients probably died of other coexisting diseases; 3) patients had detailed histopathological grade information. In the end, 337 patients in the TCGA-LIHC cohort match the above criteria, with a detailed list shown in Supplementary Tables S1, S2. Particularly, Mx denotes the uncertain status of the pathological metastasis and it ranges from M0 to M1, and Nx represents the uncertain status of the pathological nodes and it ranges from N0 to N1. A previous study (Hong, et al., 2021) merged the pathological M1 and Mx (defined as M1+Mx) and established a nomogram to predict the clinical outcomes of patients with HCC in TCGA-LIHC. Analogously, we merged patients with pathological N1 and N_X_ (defined as pathological N+), and also merged patients with pathological M1 and M_X_ (defined as pathological M+), respectively. All the 50 normal tissues were included to conduct further differential gene expression analyses. The total design of the current study was shown in Supplementary Figure S1.

### Identifying Hypoxia-Related Differential Expressed lncRNAs

We utilized the “SeqMap” software (Jiang and Wong, 2008) to re-annotate the lncRNA expression matrix in GSE155505 with the annotation file “gencode.v30. transcripts.fa” (FASTA format, 03-April-2019), publicly obtained from the “GENECODE” database (https://www.gencodegenes.org/). The analyses of differentially expressed lncRNAs (DELs) in GSE155505 and TCGA- LIHC datasets were conducted by the R “limma” package (Ritchie, et al., 2015), respectively. The criteria of DELs were set at **|**fold change**| >**1.5 and corrected *p*-value < 0.05. HRDELs were identified as the intersection of DELs in the GSE155505 and TCGA- LIHC datasets.

### Identification of HRDELs-Related HCC Clusters With Different Clinical Characteristics

All the 337 cases in the TCGA-LIHC project were unsupervisedly clustered into different groups according to the expression levels of HRDELs, using the “K-means” method in the “ConsensusClusterPlus” package. The “survival” package was employed to perform the survival analysis among different HCC clusters. Kaplan-Meier curves were plotted and the log-rank test was conducted to determine the survival difference. We further analyzed the correlation between the HRDELs-specific clusters and the corresponding clinical characteristics of each patient with HCC, including overall survival status, age, sex, Alpha-fetoprotein (AFP) level, pathological T, pathological N, pathological M, American Joint Committee on Cancer (AJCC) stage, tumor histopathological grade, and “Progressed (Yes/No)”.

### Development of the HRDELs-Derived Prognostic Signature

The prognostic signature was identified as the following steps:1) 337 cases in the entire TCGA-LIHC dataset were randomly divided into a training dataset (236 cases) and another independent testing dataset (101 cases) at the ratio of 7:3 via the R package “caret”, and particularly the testing dataset was only applied to verify the prognostic model; 2) Univariable Cox analysis was employed to select for the prognostic lncRNAs in the training dataset (*p*-value < 0.05); 3) The LASSO penalty Cox regression was employed to remove the less contributive variables via the “glmnet” package; 4) Stepwise multivariable Cox analysis was utilized to develop an optimal signature according to the minimal AIC (Akaike information criterion). The final risk score formula is defined as follows: $\;risk\;score=\overset{n}{\underset{i=1}{\sum }}expi*coefi$ , where the expi represents the expression of the specific prognostic lncRNA and the coefi represents its corresponding multivariate Cox regression coefficient.

### Evaluating and Validating the Prognostic Signature

The risk scores of HCC patients in the training dataset (236 cases), independent testing dataset (101 patients), and the entire TCGA-LIHC cohort (337 patients) were computed by the constructed formula. We split HCC patients into different hypoxia-related risk groups according to the optimal threshold value estimated by the “survminer” package in R. Survival analyses were carried out through the “survival” package, with the survival difference determined by the log-rank test. The time-dependent ROC (receiver operating characteristic) curve and the AUCs (areas under the curve) methods were employed to judge the prognostic value of the signature via the “timeROC” package.

### Relationship Between the HRDELs-Derived Signature and Clinical Characteristics

To further test the predictive ability of the HRDELs-derived signature, the overall survival difference analysis between the high-risk and low-risk group in the entire TCGA-LIHC cohort was performed using the Kaplan-Meier curve and log-rank test, according to different clinical subgroups including age (≥65 or <65 years), sex (male or female), AFP level (high ≥400 ng/ ml or low <400 ng/ ml), T (T1-2 or T3-4), M (M0 or M+), N (N0 or N+), AJCC stage (stage Ⅰ-Ⅱ or stage Ⅲ-Ⅳ), tumor histopathological grade (G1-2 or G3-4). In addition, comparisons of the distribution differences of the hypoxia-related risk groups among the different clinical characteristics were also carried out.

### Estimating the Independent Prognostic Value of the HRDELs-Derived Signature

Univariable Cox analysis and multivariable Cox analysis were carried out to identify whether the HRDELs-derived signature served as an independent prognostic factor when adjusting for other clinical parameters. We further incorporated these independent prognostic factors to construct a clinical nomogram *via* the “rms” package. Calibration curves and decision curve analysis (DCA) (Vickers and Elkin, 2006) were utilized to evaluate the calibration and clinical net benefits of the predictive model.

### GO and KEGG function enrichment analysis.

Pearson correlation was applied to explore the coexpression genes of the five key lncRNAs (*LINC00869*, *CAHM*, *RHPN1-AS1*, *MKLN1-AS*, and *DUXAP8*), according to the threshold standard of |r| > 0.3 and *p* < 0.05. Subsequently, GO and KEGG function enrichment analyses of the above coexpression genes were conducted to unravel the fundamental mechanism of the five HRDELs via the R “clusterProfiler” package (Yu, et al., 2012).

### Somatic Variant Analysis

Somatic variants profiles calculated by the “Mutect2” software in the TCGA-LIHC cohort were downloaded from the TCGA database, and the “maftools” package (Mayakonda, et al., 2018) was employed to analyze and visualize the somatic variant landscape.

### GSEA

We conducted differential gene expression analyses between the hypoxia-related high- and low-risk groups in the TCGA-LIHC cohort by the “limma” package (Ritchie, et al., 2015). All genes were ranked as a gene list according to their log2 fold change (log2FC) value. GSEA (gene set enrichment analysis) (Subramanian, et al., 2005), which calculates the enrichment score and the corresponding adjusted *p*-value of a predefined gene set according to the pre-ranked gene list based on transcriptomic expression profiles, was employed to determine the differently enriched pathways in hallmark gene sets (“h.all.v7.4. entrez.txt”) and KEGG pathways gene sets (“c2. cp.kegg.v7.4. entrez.txt”) publicly downloaded from the MsigDB database (Liberzon, et al., 2015) (http://www.gsea-msigdb.org/gsea/msigdb) via the R “clusterProfiler” package (Yu, et al., 2012). A set value of adjusted *p*-value <0.05 represents a statistical significance.

### Analyzing the Landscape of Tumor Immune Microenvironment

The single-sample gene set enrichment analysis (ssGSEA) (Yi, et al., 2020), which can estimate the relative score of a specific type of immune cell at the level of a single sample, was utilized to evaluate the relative abundance of 28 immune cells according to the specific gene signatures curated from the previously published literature (Charoentong, et al., 2017) via the R package “GSVA”. The ssGSEA is a popular bioinformatics algorithm, which was extensively utilized in cancer-related studies (Liu, et al., 2021a; Liu, et al., 2021b; Liu, et al., 2021c; Liu, et al., 2021d; Liu, et al., 2021e; Liu, et al., 2021f; Liu, et al., 2021g).

### Correlation Between HRDELs-Derived Risk score and Stemness, HIF-1A mRNA Level, and Immune Checkpoint Expression.

RNAss (RNA-based stemness scores) and DNAss (DNA methylation-based stemness scores) of HCC patients in the TCGA-LIHC cohort were publicly downloaded from the UCSC Xena database (https://pancanatlas.xenahubs.net), curated by the previously published literature (Malta, et al., 2018). Correlations between HRDELs-derived risk score and stemness, HIF-1A mRNA expression (representing the HIF-1α mRNA level), and immune checkpoint expression for each HCC patient were examined by Pearson correlation analysis, respectively.

### Prediction of Immunotherapy Responsiveness and Targeted Drug Sensitivity

Prediction of immunotherapy response in HCC patients was conducted using the TIDE (Tumor Immune Dysfunction and Exclusion) method (http://tide.dfci.harvard.edu/) (Jiang, et al., 2018). Drug sensitivities for HCC patients were estimated via the Genomics of Drug Sensitivity in Cancer (GDSC) database (Yang, et al., 2013). Drug sensitivity was assessed according to the IC_50_ (half-maximal inhibitory concentration) values of HCC patients estimated by the “pRRophetic” package (Geeleher, et al., 2014).

### Statistical Analysis

R software was employed to conduct the statistical analyses. Continual variable differences between the two groups were determined by the Wilcoxon test. Comparisons among more than two groups were performed by the Kruskal-Wallis test. The frequency differences in category variables were examined via the chi-square test or Fisher’s exact test. Survival differences were determined by the log-rank test. A threshold of two-sided *p*-value < 0.05 was set to indicate statistical significance. For multiple testing, the Benjamini–Hochberg method was employed to correct the p-value.

## Results

### Identification of the HRDELs in HCC

**FIGURE 1:**
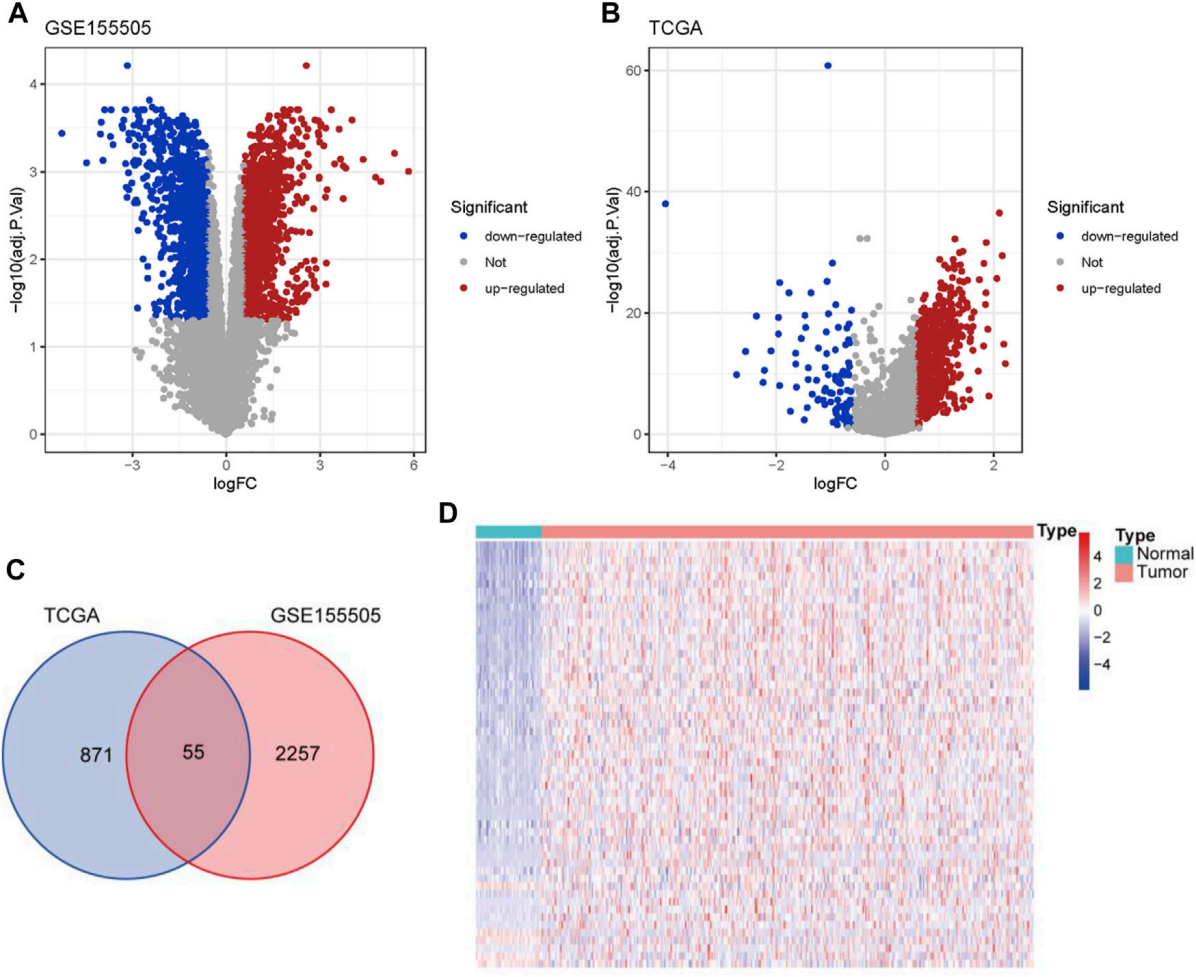
Identification of HRDELs in HCC. Volcano plots for DELs in GSE155505 **(A)** and TCGA-LIHC cohort **(B)**. **(C)** Venn diagram of hypoxia-related lncRNAs from GSE155505 and TCGA-LIHC cohort. **(D)** Heatmap of the expression levels of 55 HRDELs between HCC tumor and adjacent normal tissues in TCGA-LIHC cohort. HRDELs: hypoxia-related differentially expressed lncRNAs. DELs: differentially expressed lncRNAs. HCC: hepatocellular carcinoma. TCGA: The Cancer Genome Atlas. LIHC: liver hepatocellular carcinoma.

A previous study has established a hypoxia-related gene signature from public datasets consisting of hypoxia and normoxia HCC cells to predict the diagnosis and prognosis of HCC patients (Zhang, et al., 2020). Analogously, by conducting differential gene expression analyses between the hypoxia and normoxia HCC cells in GSE155505, we acquired 2312 DELs (**|**fold change**| >** 1.5 and adjusted p-value < 0.05) and defined them as HCC-specific hypoxia-related lncRNAs (Supplementary Table S3), including 1249 up-regulated and 1063 down-regulated lncRNAs (Figure 1A). With the same threshold criteria in the TCGA-LIHC cohort, we obtained 926 DELs (829 up-regulated and 97 down-regulated lncRNAs) in HCC tumor tissues compared with normal samples (Figure 1B; Supplementary Table S4). To further select the most contributive hypoxia-related lncRNAs in the carcinogenesis of HCC, we obtained a total number of 55 HRDELs by intersecting the HCC-specific hypoxia-related lncRNAs in GSE155505 with the DELs in TCGA-LIHC (Figure 1C; Supplementary Table S5). Of note, the majority of those HRDELs possessed elevated expression levels not only in hypoxia-treated HCC cells in GSE155505 (Supplementary Figure S2) but also in the HCC tumor tissues in TCGA-LIHC (Figure 1D), indicating that the above 55 HRDELs substantially contribute to the tumorigenesis of HCC.

### Microdissection of the HRDELs-Related Clusters in HCC

**FIGURE 2:**
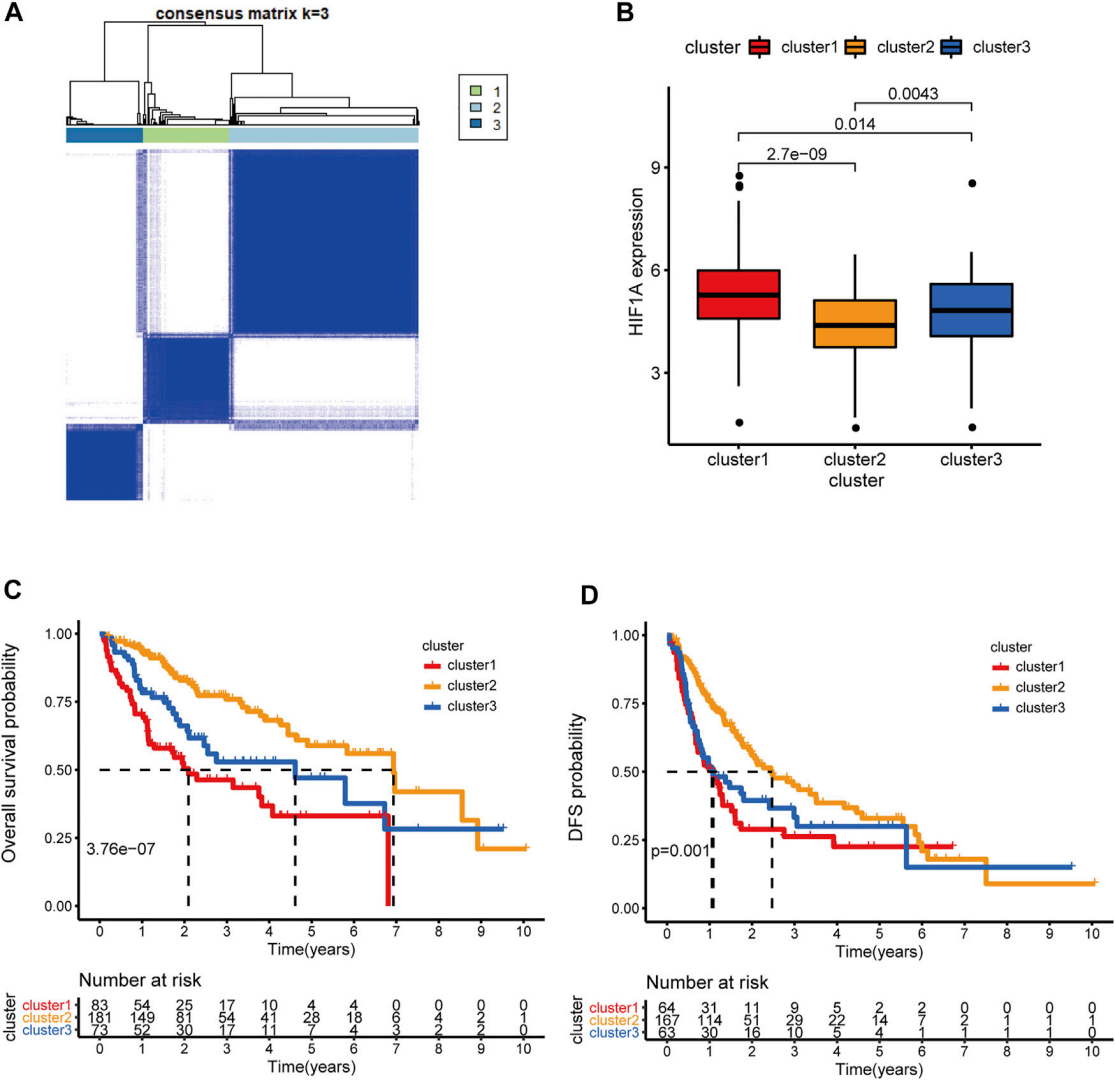
Microdissection of the hypoxia-related lncRNA landscape in TCGA-LIHC cohort. **(A)** the Consensus matrix plot of HCC patients by unsupervised clustering (K-means method) according to the expression levels of 55 HRDELs, when k = 3 representing the optimal cluster number. **(B)** Comparison of HIF1A mRNA expression among the HRDEL-specific clusters. **(C)** Overall survival difference and **(D)** DFS difference among hypoxia-specific clusters. HCC: hepatocellular carcinoma. HRDELs: hypoxia-related differentially expressed lncRNAs. DFS: disease-free survival.

The HRDELs landscape in patients with HCC in the TCGA-LIHC cohort was microdissected by unsupervised clustering according to the expression levels of the 55 aforementioned HRDELs, via the “K-means” algorithm in the “ConsensusClusterPlus” package. We selected 3 as the optimal k value because that the k value of 3 could simultaneously possess a high cumulative distribution function (CDF) value and a clear separation of the consensus matrix (as shown in Figure 2A; Supplementary Figures S3A–D). Therefore, all cases were assigned into three groups according to the unsupervised clustering results (Figure 2A). In brief, cluster 1, cluster 2, and cluster 3 include 83, 181, and 73 cases, respectively (Supplementary Table S6). Cluster2 showed the lowest mRNA expression level of HIF1A compared with cluster 1 (Figure 2B, *p* = 2.7e−09) and cluster 3 (*p* = 0.0043). Notably, there were significant OS differences among the three clusters (Figure 2C, global *p* = 3.76e−07). Cluster 2 possessed a longer median OS time than cluster 1 (*p* = 2.107e−08) and cluster 3 (*p* = 0.011), while there was no significant OS difference between cluster 1 and cluster 3 (*p* = 0.051). Survival analysis also showed that cluster 2 exhibited better disease-free survival (DFS) outcomes (Figure 2D, global *p* = 0.001) than cluster 1 (*p* = 4.41e-04) and cluster 3 (*p* = 0.015), whereas no statistical significance was shown between cluster 1 and cluster 3 (*p* = 0.493). These results indicate that cluster 2 with the lowest HIF1A mRNA expression level represents the least hypoxic exposure in HCC and has the best survival outcomes. Thus, we conclude that the hypoxia-related lncRNA landscape indeed correlates with the clinical outcomes of HCC patients.

### Clinical Correlation Analysis of HRDELs-Related Clusters

**FIGURE 3:**
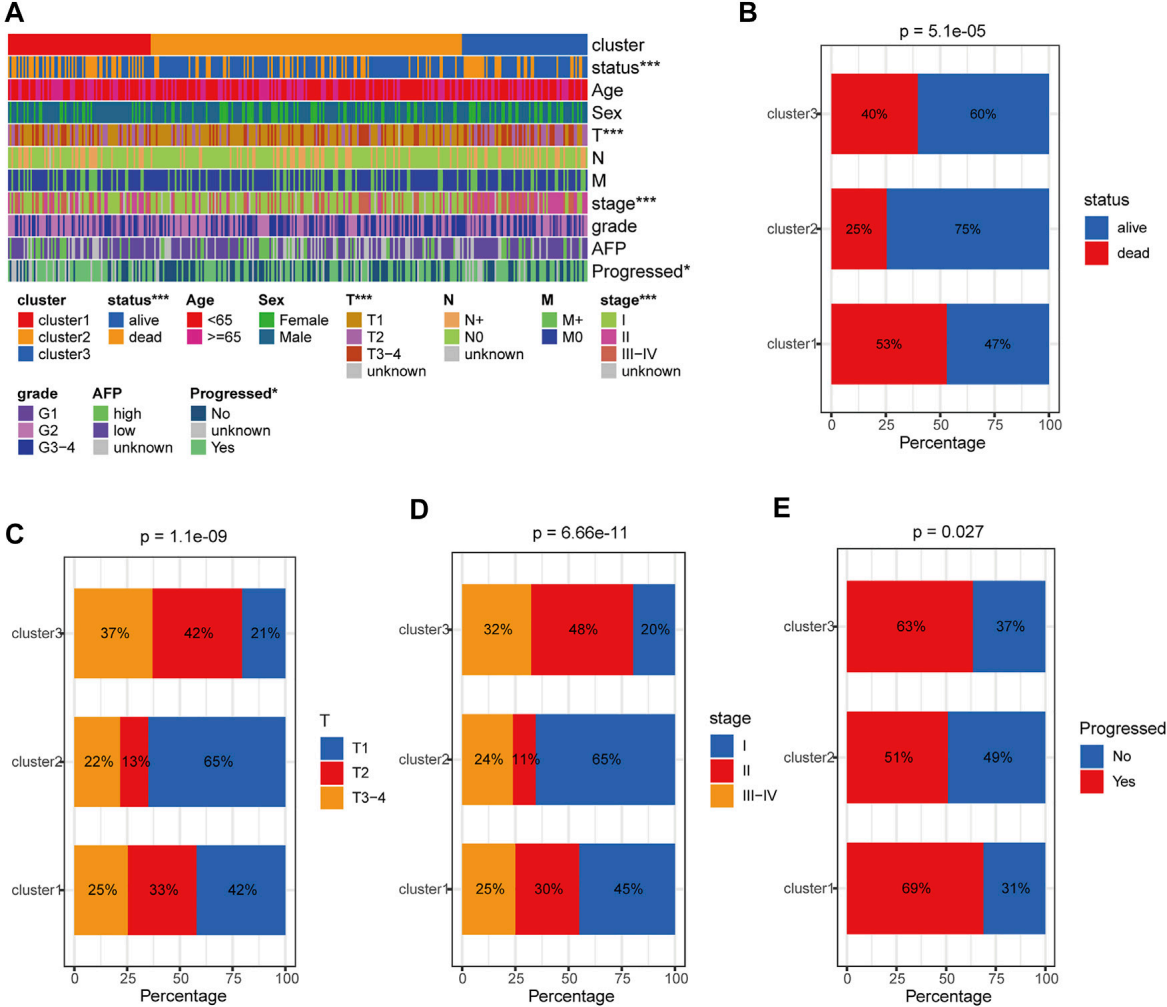
Clinical correlation analysis of HRDELs-specific clusters in TCGA-LIHC cohort. **(A)** Distribution landscape of HRDELs-specific clusters among clinical characteristics. Comparison of distribution difference of overall survival status **(B)**, pathological T **(C)**, AJCC stage **(D)**, and “Progressed (Yes/No)” **(E)** among HRDELs -specific clusters. HRDELs: hypoxia-related differentially expressed lncRNAs. AJCC: American Joint Committee on Cancer. ***, *p* < 0.001; **, *p* < 0.01; *, *p* < 0.05.

We further comprehensively analyzed the association of the HRDELs-related clusters and clinical characteristics in the TCGA-LIHC cohort. Results showed that there were significant distributive differences in overall survival status, pathological T, AJCC stage, and “Progressed (Ye/No)” among HRDELs-related clusters (Figure 3A). Cluster 2 has a lower death rate of patients with HCC (25%) compared to cluster1 (53%), and cluster3 (40%), as shown in Figure 3B (*p* = 5.1e−05). Cluster 2 had a higher proportion of patients with pathological T1 (65%), stage Ⅰ (65%), and “Progressed (No) (49%)” than cluster 1 (42, 45, and 31%, respectively) and cluster 3 (21, 20, and 37%, respectively), as shown in Figures 3C–E. The above evidence suggests that HRDELs-related clusters are closely associated with tumor progression in HCC.

### Construction of the HRDELs-Derived Prognostic Signature

**FIGURE 4:**
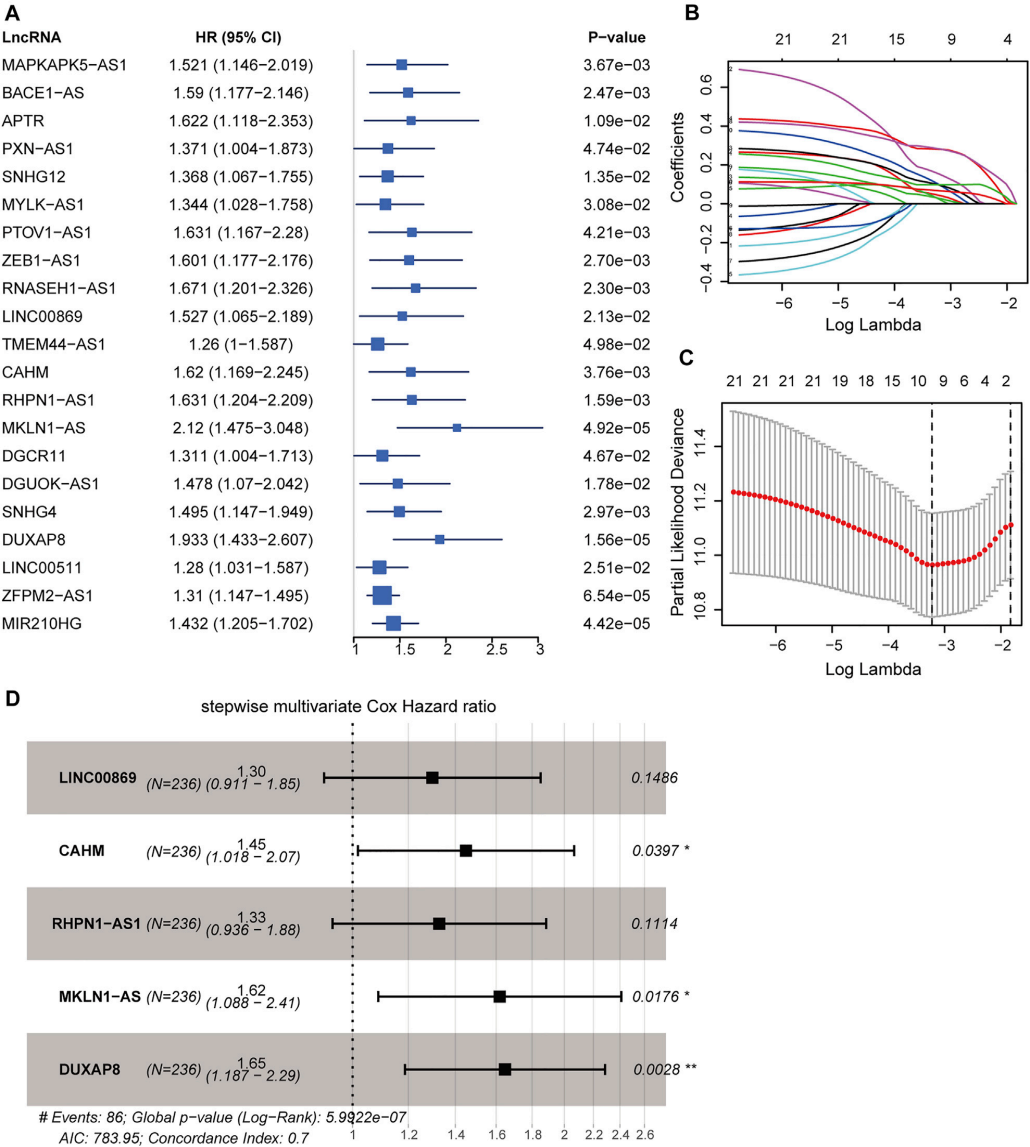
Construction of hypoxia-related lncRNA signature in the training dataset. **(A)** Forest plot of 21 significant prognostic lncRNAs determined by the univariate Cox regression. **(B)** LASSO penalty coefficients of the above 21 prognostic lncRNAs. **(C)** Cross-validation of the LASSO Cox regression model, the left vertical dashed line represents the “lambda. min” standard. **(D)** Forest plot of the optimal model determined by the stepwise multivariate Cox analysis according to the minimal AIC value (783.95). LASSO: least absolute shrinkage and selection operator. AIC: Akaike information criterion. ***, *p* < 0.001; **, *p* < 0.01; *, *p* < 0.05.

All 337 patients in the TCGA-LIHC cohort were randomly assigned into the training dataset (236 cases) and the testing dataset (101 cases). The prognostic signature was developed in the training dataset. We utilized the univariable Cox regression to yield 21 significant prognostic hypoxia-related lncRNAs (Figure 4A). Subsequently, 10 prognostic lncRNAs were retained after filtering the variables by LASSO penalty Cox analysis according to the “lambda. min” standard (Figures 4B,C; Supplementary Table S7). Furthermore, the stepwise multivariable Cox regression model was employed to establish the optimal signature (Figure 4D; Supplementary Table S8). Ultimately, five hypoxia-related lncRNAs were selected and incorporated into the final model: risk score = 0.26120*LINC00869 expression+0.37141*CAHM expression+0.28394*RHPN1-AS1 expression +0.48183* MKLN1-AS expression +0.49900*DUXAP8 expression.

### Evaluating and Validating the Performance of the Prognostic Signature

**FIGURE 5:**
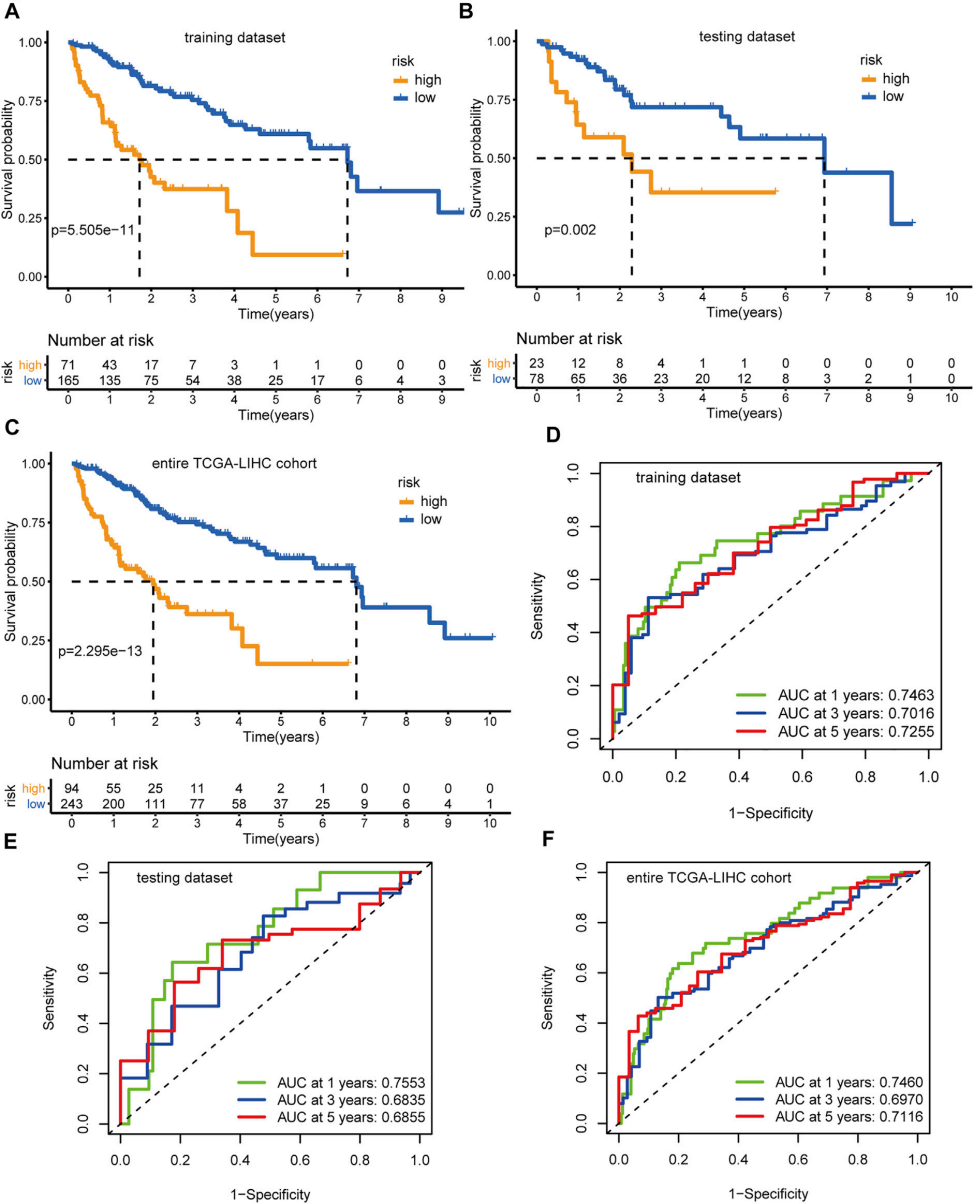
Identification and validation of the hypoxia-related lncRNAs signature. Kaplan-Meier curves and log-rank test p-value of the training dataset **(A)**, testing dataset **(B)**, and entire TCGA-LIHC cohort **(C)**, respectively. The AUCs of the time-dependent ROC curves for the training dataset **(D)**, testing dataset **(E)**, and entire TCGA-LIHC cohort **(F)**, respectively.

Applying the above formula, we computed the hypoxia-related risk score for each patient in the training dataset (Supplementary Table S9). All these cases were assigned into a high-risk (71 patients) or low-risk group (165 patients) based on the optimal threshold value (2.3033). The high-risk group showed an adverse prognosis compared with those in the low-risk counterpart (*p* < 0.001, Figure 5A). The AUCs of the risk scores for the 1-, 3-, and 5-years survival predictions were 0.746, 0.702, and 0.726 (Figure 5D), respectively, indicating a good predictive value. We further tested the prognostic model in the testing dataset (Supplementary Table S10) and the entire TCGA-LIHC dataset. With the same threshold, cases in the testing dataset and the entire TCGA-LIHC dataset were assigned into different hypoxia-related risk groups, respectively. Analogously, the high-risk group consistently showed a poorer clinical outcome than the low-risk group, with *p* = 0.002 in the testing dataset (Figure 5B) and *p* < 0.001 in the entire TCGA-LIHC dataset (Figure 5C), respectively. The AUCs for the 1-, 3-, and 5-years prognostic prediction in the testing dataset were 0.755, 0.684, and 0.686, respectively (Figure 5E), and the AUCs of the entire TCGA-LIHC cohort were 0.746, 0.697, and 0.712 for 1-, 3-, and 5- year survival prediction, respectively (Figure 5F). These results demonstrate the robustness and reliability of the prognostic signature.

We further sought to search for an external validation dataset in the International Cancer Genome Consortium (ICGC) database or GEO database, but unfortunately, there was no other public dataset of HCC patients with matched lncRNA expression profiles and complete survival information. Finally, we chose the HCC dataset GSE14520-GPL3921 as the external validation dataset and re-annotated all the probe sequences using the “SeqMap” software to obtain the lncRNA expression profiles. However, only three lncRNAs (LINC00869, RHPN1-AS1, and MKLN1-AS) in the hypoxia-related lncRNA signature were re-annotated in GSE14520-GPL3921 and thus we had to calculate the risk score through the following formula: risk score = 0.26120*LINC00869 expression+0.28394*RHPN1-AS1 expression + 0.48183* MKLN1-AS expression. GSE14520-GPL3921 comprises 225 HCC tissues and 220 non-tumor specimens, and 221 tumor samples with detailed survival data were enrolled as the validation dataset. We calculated the risk score for each HCC patient (Supplementary Table S11) and categorized patients into different risk groups based on the optimal threshold (9.753). In the same manner, Kaplan-Meier curves demonstrated that patients in the high-risk group had poorer clinical outcomes than those in the low-risk counterpart (Supplementary Figure S4A, *p* = 0.032). ROC analyses showed that The AUCs for the 1-, 3-, and 5-years prognosis prediction were 0.510, 0.570, and 0.534, respectively (Supplementary Figure S4B). The unsatisfactory AUC values in GSE14520-GPL3921 might be caused by the lack of expression profiles of CAHM and DUXAP8, and further complete external validation will still be needed in the future. Collectively, the external validation results further confirmed that the hypoxia-related lncRNA signature was closely associated with adverse clinical outcomes in HCC.

### Subgroup Survival Analysis of the HRDELs-Derived Signature

We further stratified the entire TCGA-LIHC cohort into different subgroups according to the clinical characteristics including age (≥65 or <65 years), sex (male or female), AJCC stage (stage Ⅰ-Ⅱ or stage Ⅲ-Ⅳ), pathological T (T1-2 or T3-4), pathological M (M0 or M + ), pathological N (N0 or N+), tumor histopathological grade (G1-2 or G3-4), AFP level (high ≥400 ng/ml or low <400 ng/ ml). Strikingly, patients with high-risk scores consistently had poorer clinical outcomes than those with low-risk scores, no matter which subgroups they are in (Supplementary Figures S5–S7). This further confirms the reliable prognostic value of the hypoxia-related lncRNA signature in predicting the clinical outcomes of patients with HCC.

### Identifying the Independent Prognostic Value of Hypoxia-Related lncRNA Signature

**FIGURE 6:**
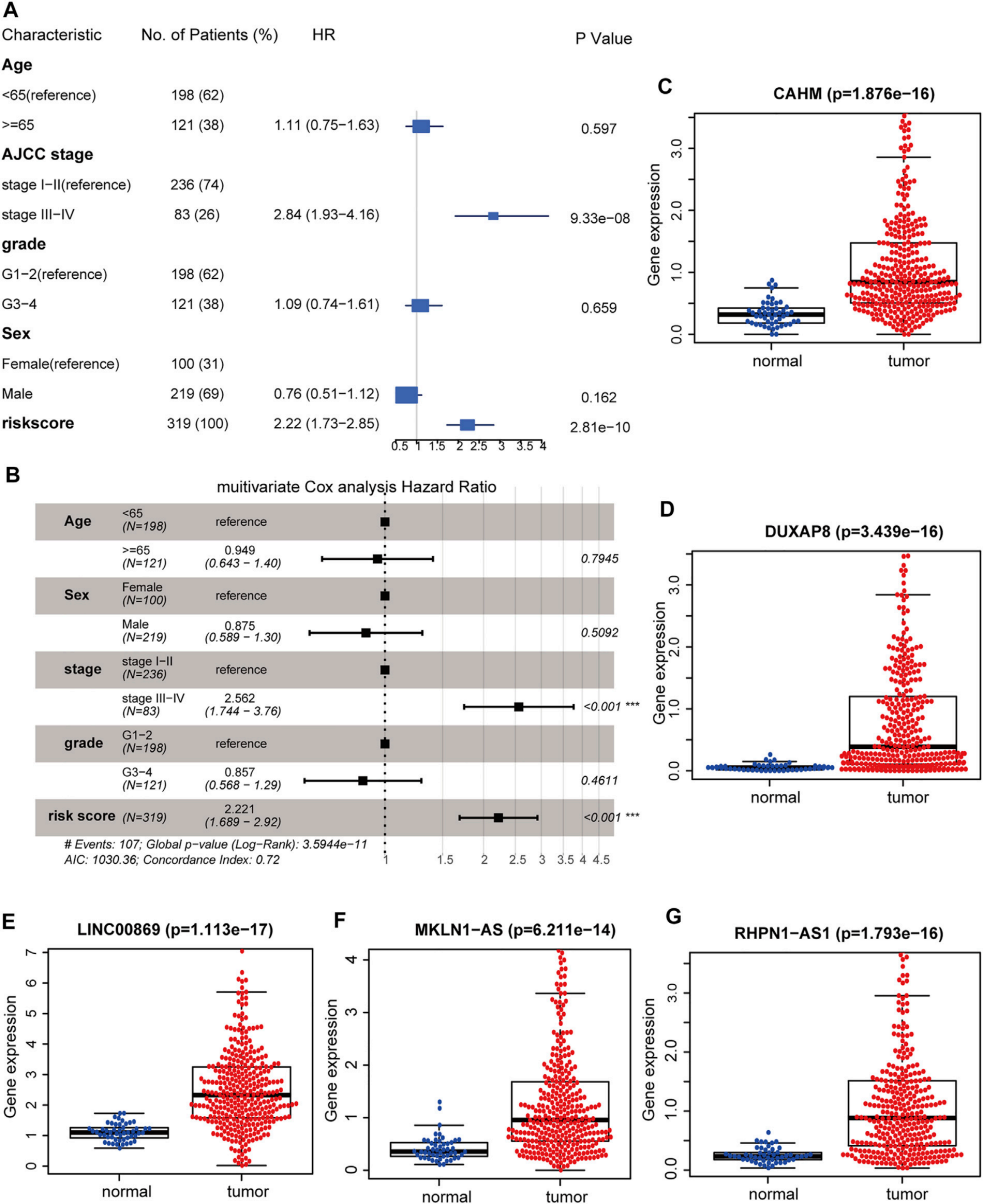
Identifying the hypoxia-related lncRNA signature as an independent prognostic factor. Forest plot of the corresponding p-values of the univariate Cox regression analysis **(A)** and multivariate Cox regression analysis **(B)**. Comparisons of the expression levels of CAHM **(C)**, DUXAP8 **(D)**, LINC00869 **(E)**, MKLN1-AS **(F)**, and RHPN1-AS1 **(G)** between HCC tumor and adjacent normal tissues in TCGA-LIHC cohort. TCGA: The Cancer Genome Atlas. LIHC: liver hepatocellular carcinoma. ***, *p* < 0.001; **, *p* < 0.01; *, *p* < 0.05.

**FIGURE 7:**
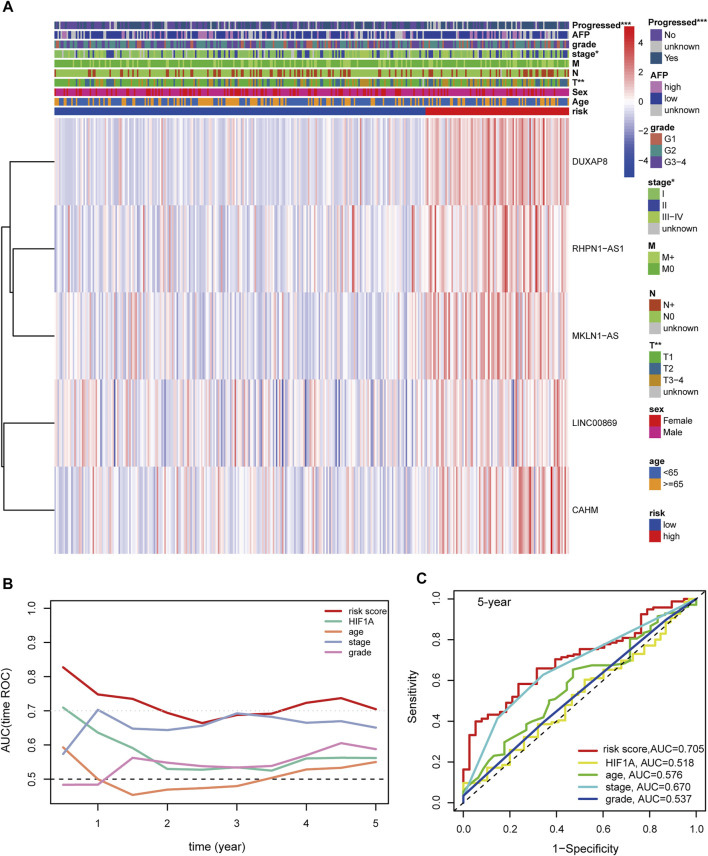
**(A)** Distribution landscape of the hypoxia-related risk groups among clinical parameters and the heatmap of the expression levels of the five key lncRNAs in HCC patients in the TCGA-LIHC cohort. The color blue denotes a low expression level and red represents a high expression level. **(B)** AUCs of the time-dependent ROC curves for risk score, HIF1A mRNA expression, age, stage, and tumor grade in HCC patients. **(C)** AUCs for the 5-years prognostic prediction of risk score, HIF1A mRNA expression, age, stage, and tumor grade in HCC patients. AUC: area under the curve. ROC: receiver operating characteristic curve. HCC: hepatocellular carcinoma. ***, *p* < 0.001; **, *p* < 0.01; *, *p* < 0.05.

Univariable and multivariable Cox analyses consistently demonstrated that hypoxia-related risk scores and the AJCC stage were independent prognostic indicators in HCC (Figures 6A,B). Moreover, the risk score was tightly associated with pathological T, AJCC stage, and “Progressed (Yes/No)” (Figure 7A). The high-risk group has a higher proportion of patients with T3-4, stage Ⅲ-Ⅳ, and “Progressed (Yes)” than the low-risk counterpart (Supplementary Figures S8A–C). Time-dependent ROC illustrates that the 5-years AUC of hypoxia-related risk scores for the prognostic prediction reaches 0.705 and is superior to that of age, AJCC stage, pathological grade, and HIF1A mRNA expression (Figures 7B,C), indicating the good performance of the hypoxia-related lncRNA signature. Furthermore, The five lncRNAs in the prognostic signature (*CAHM*, *DUXAP8*, *LINC00869*, *MKLN1-AS*, and *RHPN1-AS1*), all had a significantly higher expression level in HCC tumor samples than normal samples in the TCGA-LIHC cohort (Figures 6C–G), implying that they probably act as oncogenic lncRNAs in the tumorigenesis of HCC.

### Construction of a Clinical Nomogram to Improve Prognostic Prediction

**FIGURE 8:**
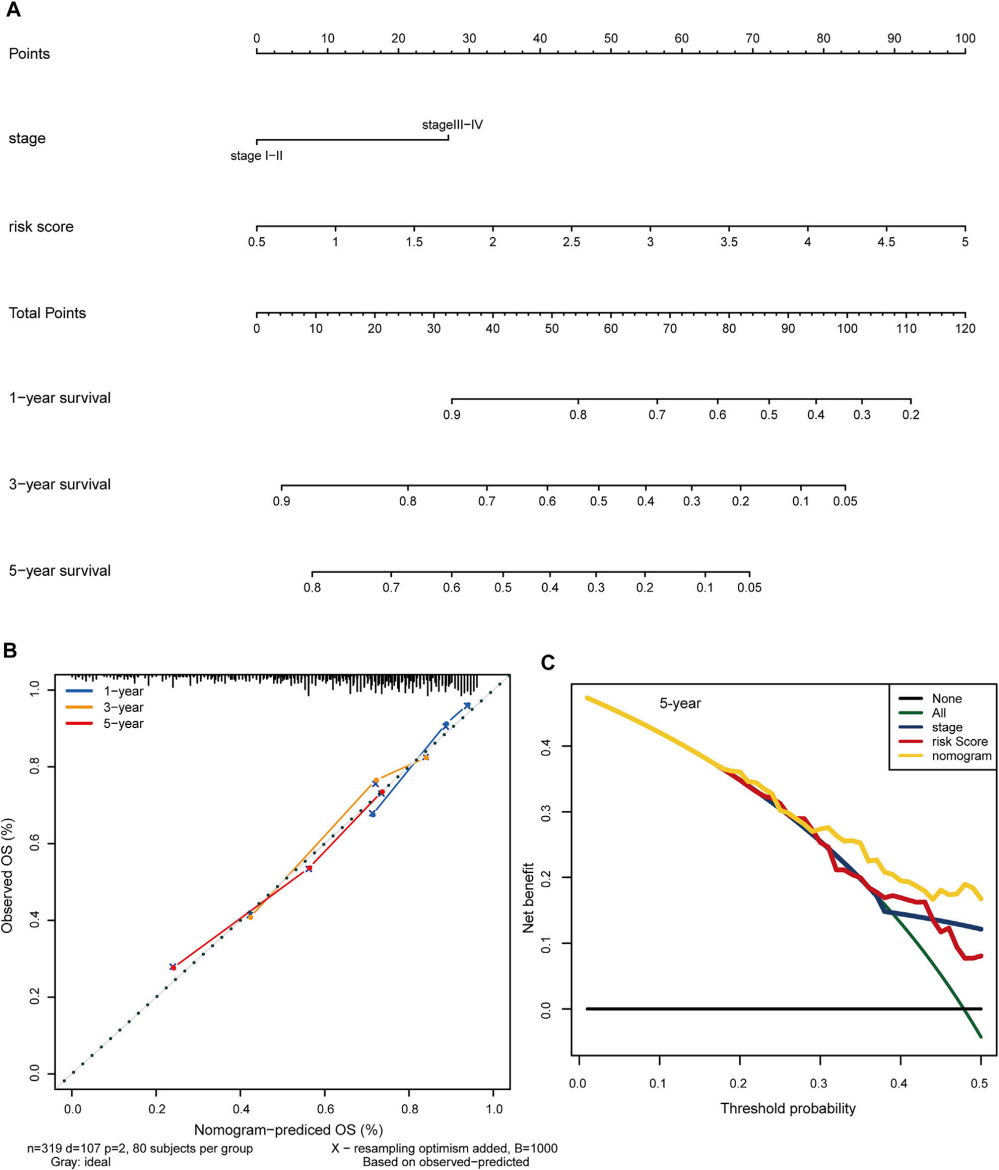
Construction of a clinical predictive nomogram to improve the prognostic prediction in HCC. **(A)** The hybrid nomogram combining the hypoxia-related risk score with the AJCC stage. Patients were given a total risk score based on each factor level in the nomogram. **(B)** Calibration curves show the consistency between the nomogram-predicted OS probability and the observed OS probability. **(C)** DCA curves illustrate the 5-years clinical net benefit of the combined nomogram compared with other individual models. HCC: hepatocellular carcinoma. OS: overall survival. DCA: decision curve analysis.

To test the clinical practicability of the hypoxia-related lncRNA signature, the two independent prognostic indicators yielded by the multivariable Cox analyses, hypoxia-related risk score and AJCC stage, were incorporated to develop a hybrid nomogram to facilitate the prognostic prediction. Patients were given a total risk score based on each factor level in the nomogram (Figure 8A). Statistical analysis showed that the concordance index (C-index) of the nomogram reached 0.718 (95% confidence interval: 0.666–0.770). Calibration curves showed that the nomogram-predicted OS probability was consistent with the observed OS probability (Figure 8B). DCA curves further suggested that the 5-years clinical net benefit of the combined nomogram was superior to that of other individual models (Figure 8C).

### Functional Annotation of Five Key Prognostic lncRNAs in HRDELs-Derived Signature

**FIGURE 9:**
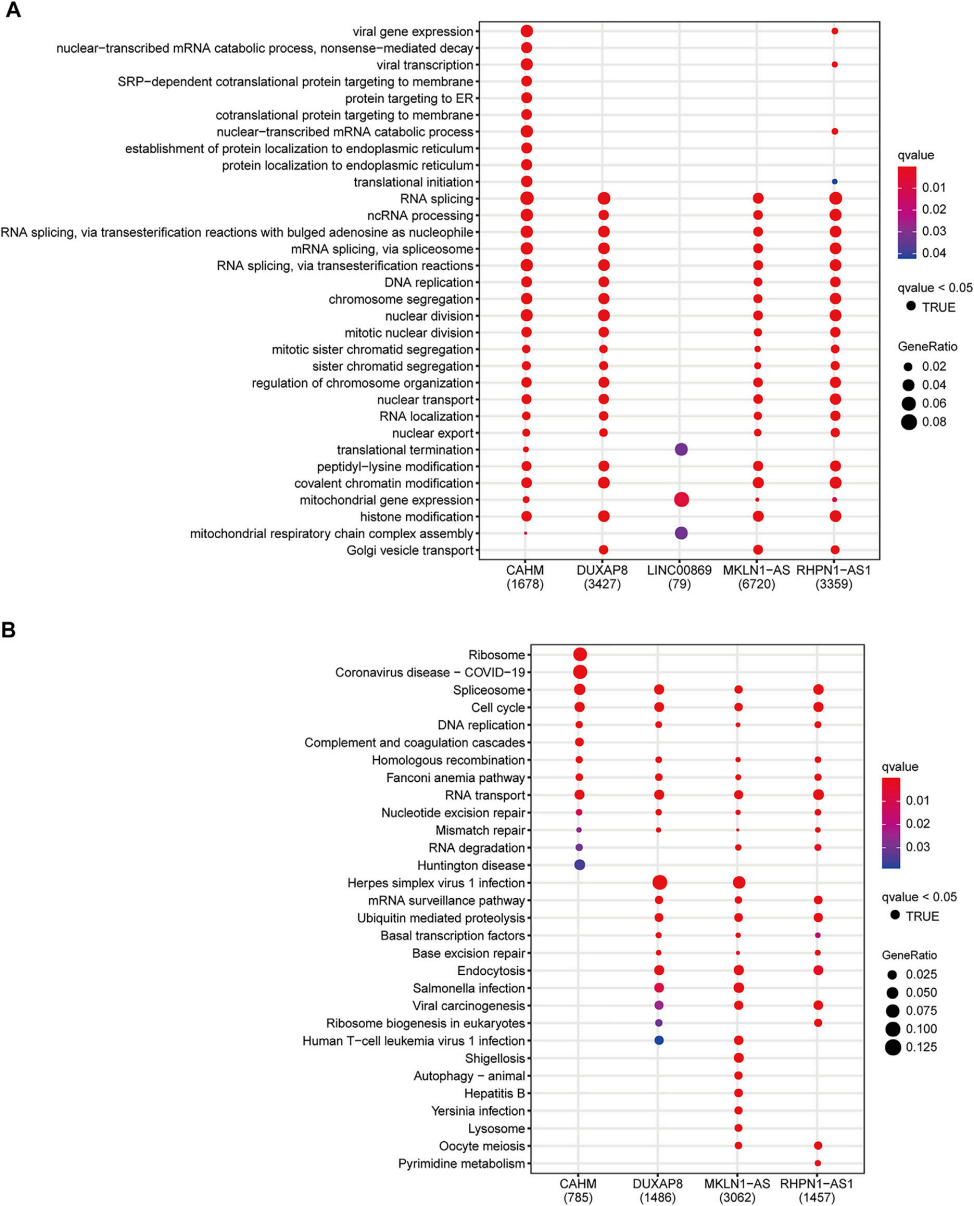
Functional annotation of CAHM, DUXAP8, LINC00869, MKLN1-AS, and RHPN1-AS1. Significantly enriched terms in the GO biological process terms **(A)** and KEGG pathway **(B)**, according to the corresponding coexpression genes of the above five key lncRNAs. GO: Gene Ontology. KEGG: Kyoto Encyclopedia of Genes and Genomes.

To investigate the underlying mechanism of the signature, we used the Pearson correlation analysis to select potential targeted genes of the five key lncRNAs. We finally obtained 1678, 3427, 79, 6720, and 3359 coexpression genes for CAHM, DUXAP8, LINC00869, MKLN1-AS, and RHPN1-AS1, respectively (|r| > 0.3 and *p* < 0.05). These corresponding coexpression genes for each key lncRNA were subjected to GO and KEGG function enrichment analysis. With the GO biological process (BP) term enrichment, four of the five key lncRNAs except for LINC00869 were consistently enriched in the tumor proliferation process including DNA replication, RNA splicing, nuclear division, mitotic nuclear division, and nuclear transport (Figure 9A). We also noticed that LINC00869 had a significant enrichment in “mitochondrial gene expression,” and “mitochondrial respiratory chain complex assembly” (Figure 9A), suggesting that LINC00869 was closely related to mitochondrial energy metabolism. For the KEGG pathway, CAHM, DUXAP8, MKLN1-AS, and RHPN1-AS1 were all enriched in these tumor proliferation-related pathways such as Spliceosome, Cell cycle, DNA replication, and RNA transport (Figure 9B), suggesting their important role in the tumorigenesis. However, there was no significantly enriched KEGG pathway associated with LINC00869. Owing to the fewer coexpression genes for LINC00869 in HCC tissues, we further compared the expression level of LINC00869 between the HCC tumor samples and non-tumor samples in GSE14520-GPL3921. Notably, LINC00869 also possessed a significantly higher expression level in tumor tissues in comparison with non-tumor tissues (Supplementary Figure S8D, *p* = 4.964e−20), confirming the critical role of LINC00869 in the carcinogenesis of HCC. We speculate that the reason for the fewer coexpression genes may be due to the unique expression pattern and molecular mechanism of LINC00869, and this phenomenon is worth further study.

### Distinct Molecular Patterns Among the Hypoxia-Related Risk Groups

On account of the significant survival difference between the two groups, GSEA was performed to elucidate the underlying molecular mechanism. With the hallmark gene sets, the high-risk group possessed significantly enriched scores in the “G2M_CHECKPOINT,” “MITOTIC_SPINDLE,” “PI3K_AKT_MTOR_SIGNALING,” “WNT_BETA_CATENIN_SIGNALING,” and “EPITHELIAL_MESENCHYMAL_TRANSITION” pathways which were strongly associated with tumor cell proliferation and aggression (Supplementary Figure S9A). In particular, the “HALLMARK_HYPOXIA” pathway was also significantly enriched in the high-risk cohort, confirming a strong correlation between the hypoxia-related lncRNA signature and hypoxic exposure in HCC. In the case of the KEGG pathway gene sets, the high-risk cohort displayed significantly enriched scores in the “CELL_CYCLE,” “SPLICEOSOME,” “PATHWAYS_IN_CANCER,” and “ADHERENS_JUNCTION” pathways (Supplementary Figure S9B). Furthermore, the high-risk cohort showed a higher level of the RNAss (RNA-based stemness scores), DNAss (DNA methylation-based stemness scores), and HIF1A expression level compared with the low-risk counterpart (Supplementary Figures S10A–C). The hypoxia-related risk score also had a significant positive correlation with RNAss, DNAss, and the HIF1A mRNA expression level (Supplementary Figures S10D–F), supporting the pivotal role of hypoxia in promoting the stemness in HCC. Collectively, the hypoxia-related lncRNA signature indeed reflects the hypoxic exposure in HCC, and hypoxia-related lncRNAs also contribute to the stemness and tumor progression of HCC.

### Somatic Variants Analysis

**FIGURE 10:**
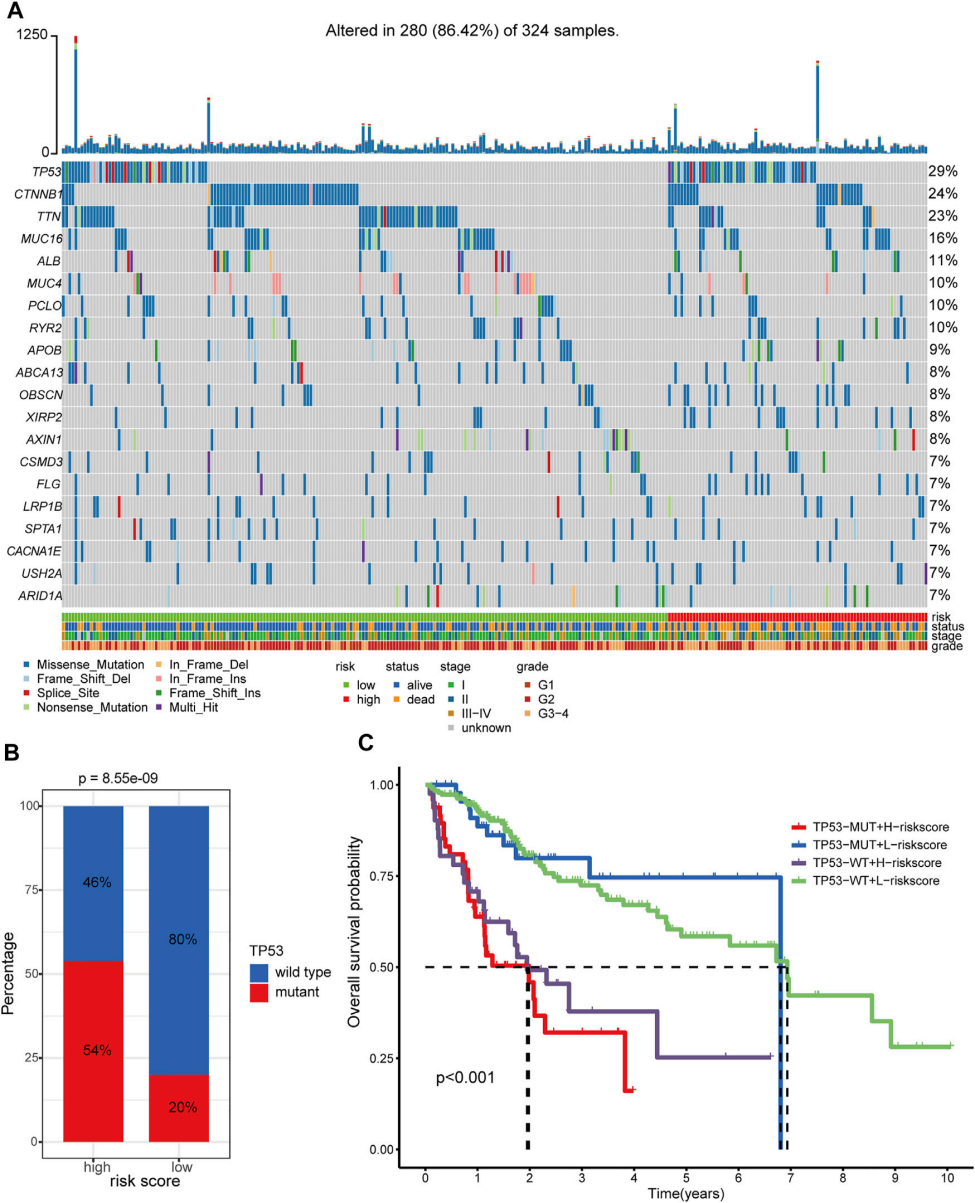
Somatic variants analysis of patients in TCGA-LIHC cohort. **(A)** Somatic variants landscape of the top 20 frequently mutational genes in the two risk groups. **(B)** Comparison of mutational frequency differences of TP53 between hypoxia-related high-risk and low-risk groups. **(C)** Survival analyses of the different clinical subgroups stratified by TP53 status and hypoxia-related risk score. TP53-MUT: TP53-mutant. TP53-WT: TP53-wild type. H-risk score: high-risk score. L-risk score: low-risk score.

In total, we obtained the somatic variants profiles of 324 HCC patients enrolled in our study by matching the patient identity number. The distributive landscape of the top 20 frequently mutated genes between the two groups was depicted in Figure 10A, and *TP53*, *CTNNB1*, and *TTN* ranked as the top three mutative genes. Studies have reported that mutant *TP53* can cooperate with hypoxia to promote tumor progression (Amelio, et al., 2018; Zhang, et al., 2021). Thus, we focus on the relationship between the *TP53* mutational status and the hypoxia-related lncRNA signature. The Chi-square test showed that *TP53* had a significantly higher mutative ratio in the high-risk group than in the low-risk counterpart (54 versus 20%, *p* = 8.55e−09, Figure 10B). With respect to the comparisons of the mutative ratio of *CTNNB1* and *TTN,* there was no significant difference between the two groups (Supplementary Figures S11A–B). Subgroup survival analysis further indicated that patients with low-risk scores consistently had better OS survival outcomes than those with high-risk scores irrespective of the *TP53* status (Figure 10C, global p-value < 0.001). Moreover, patients with a wild type of *TP53* in the high-risk or low-risk group showed better clinical outcomes than patients with a mutant type of *TP53* in the corresponding group. In the case of *CTNNB1* and *TTN,* subgroup survival analyses showed the same results as *TP53* (Supplementary Figures S11C–D). These results support that hypoxia contributes to genome instability and the crosstalk between these frequently mutated genes (*TP53, CTNNB1,* and *TTN*) and hypoxia has a substantial impact on the prognosis of patients with HCC.

### Correlation Between the Hypoxia-Related lncRNA Signature and Tumor Immune Microenvironment

**FIGURE 11:**
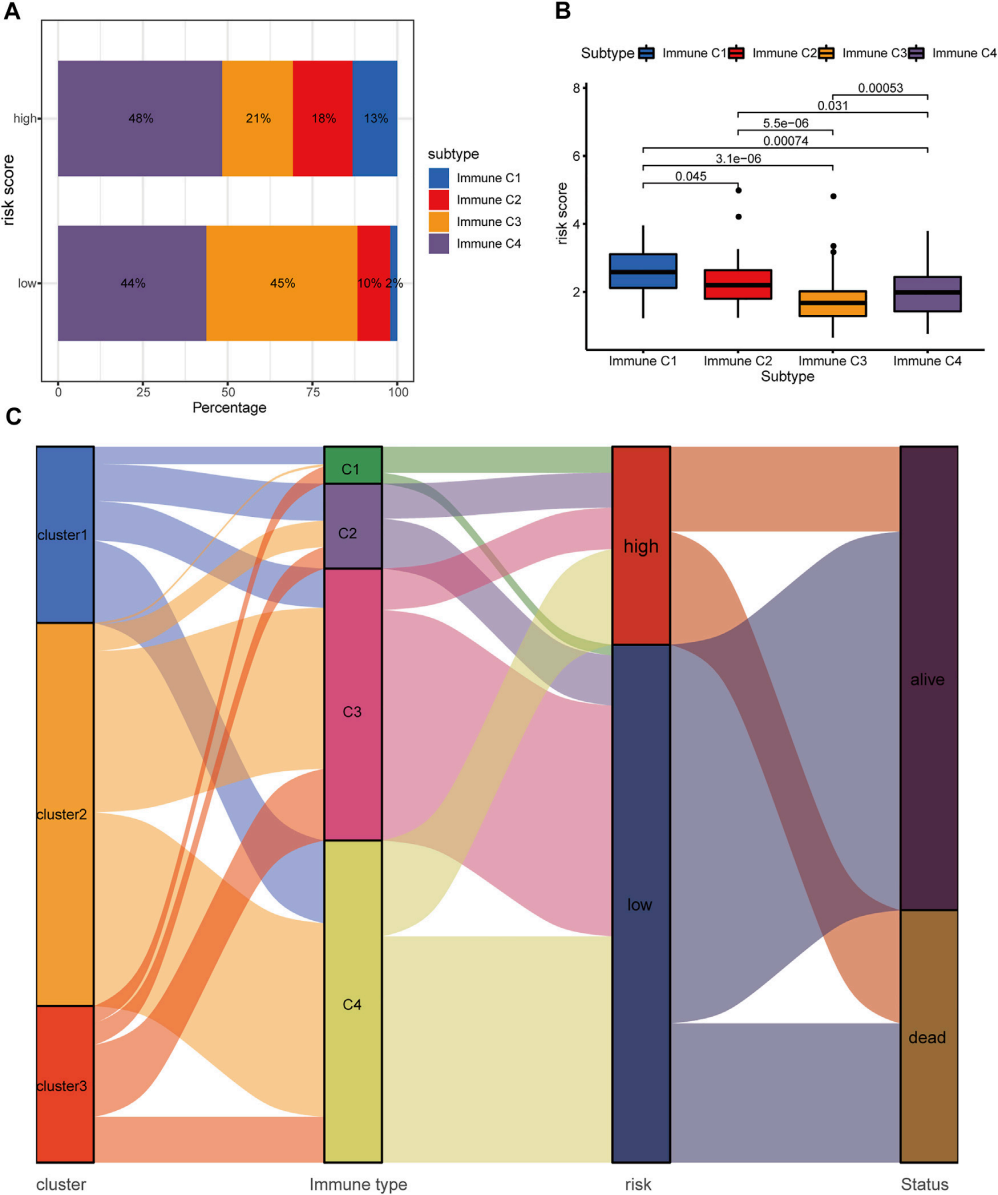
Correlation between the hypoxia-related lncRNA signature and classical immune subtypes. **(A)** Comparison of the distributive difference of the immune subtypes between the two risk groups. **(B)** Comparisons of the hypoxia-related risk scores among different immune subtypes. **(C)** The alluvial plot illustrating the relationship between the HRDELs-specific clusters, classical immune subtypes, hypoxia-related risk groups, and overall survival status. HRDELs: hypoxia-related differentially expressed lncRNAs.

A previously published study has already classified more than 10,000 tumor samples across 33 cancer types in TCGA into six classical immune subtypes (immune C1, C2, C3, C4, C5, and C6) and found that patients in the immune type C3 (inflammatory type) have the best survival outcomes (Thorsson, et al., 2018). Thus, we further investigated the association of the hypoxia-related lncRNA signature and the classical immune subtypes. In total, 330 out of the 337 HCC patients in our study matched the immune subtype information (17, 39, 125, 148, and 1 patient for immune C1, C2, C3, C4, and C6, respectively). We excluded the immune C6 with only one patient from further analysis to avoid potential bias. Fisher’s exact test revealed that the low-risk group had a significantly higher proportion of immune C3 than the high-risk group (45 versus 21%, *p* = 2.7 e−06, Figure 11A). Furthermore, the immune C3 showed the lowest risk scores compared with other immune subtypes (Figure 11B). The alluvial plot showed that the immune C3 was mainly derived from HRDELs-specific cluster 2 and the majority of immune C3 was attributed to the low-risk group which had a favorable prognosis in HCC (Figure 11C). These results indicated that the hypoxia-related low-risk group had a different tumor immune infiltration pattern from the high-risk group.

**FIGURE 12:**
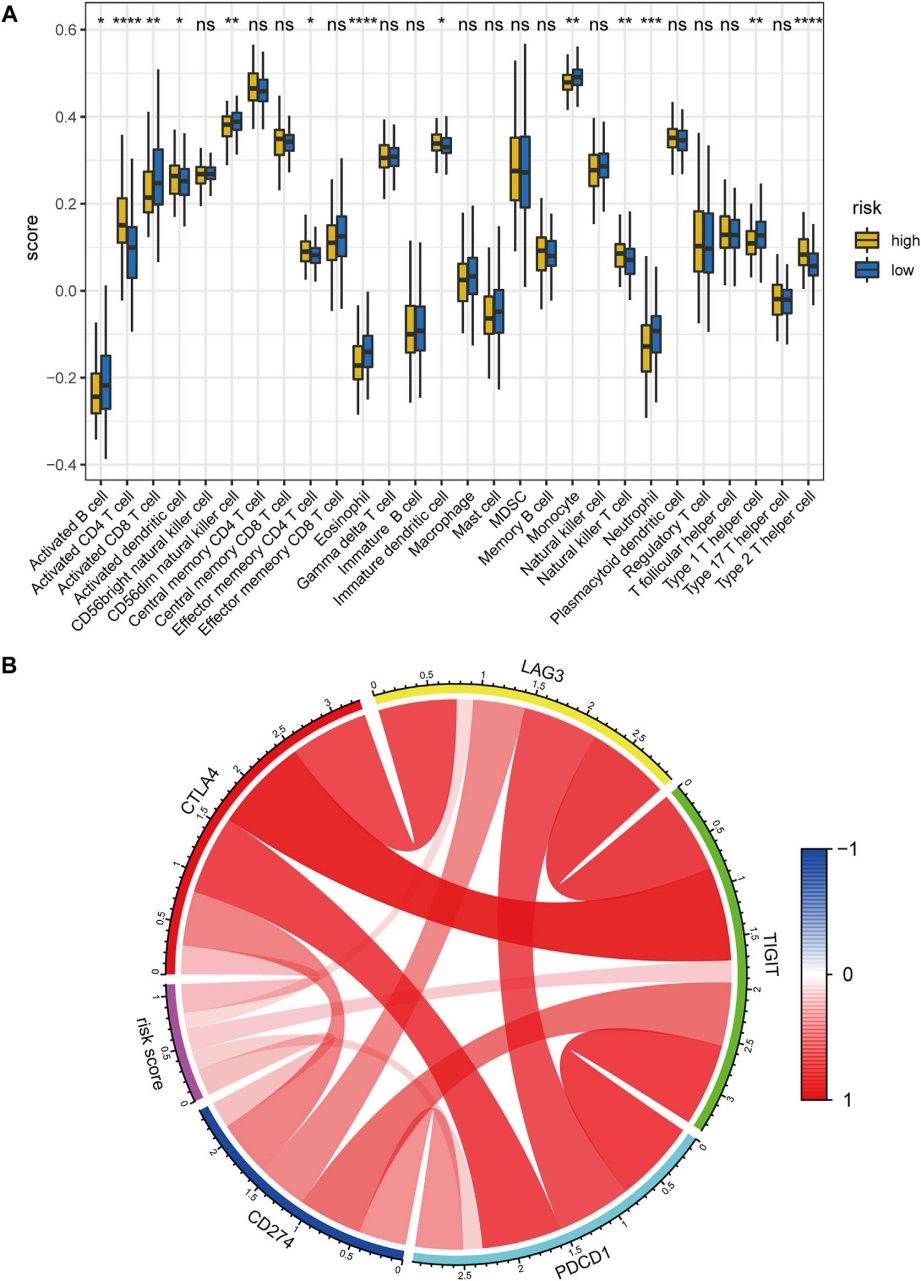
Correlation between the hypoxia-related lncRNA signature and tumor immune microenvironment. **(A)** Comparisons of the abundance of 28 immune cells between the high- and low-risk group using ssGSEA. **(B)** Chord diagram of the correlation between hypoxia-related risk score and the expression levels of PD1(PDCD1), PDL1(CD274), CTLA4, LAG3, and TIGIT. The color red denotes the positive correlation and blue represents the negative correlation. ssGSEA: single-sample gene set enrichment analysis. ****, *p* < 0.0001; ***, *p* < 0.001; **, *p* < 0.01; *, *p* < 0.05; ns: no significance.

We then calculated the relative scores of 28 immune cells for each patient with HCC using the ssGSEA algorithm (Supplementary Table S12, detailed method is described in the “Materials and methods” part). Notably, The low-risk group possessed a higher abundance in activated CD8^+^ T cell, activated B cell, monocyte, neutrophil, while the high-risk group had a higher fraction in activated CD4^+^ T cell and immature dendritic cell, and activated dendritic cell (Figure 12A). We further explored the correlation between the abundance of 28 immune cells and the expression levels of the five key lncRNAs in the hypoxia-related lncRNA signature by Pearson correlation analysis (Supplementary Figure S12). Interestingly, MKLN1−AS was significantly positively correlated with several types of immune cells such as Activated CD4 T cell, Immature dendritic cell, Effector memory CD4 T cell, Plasmacytoid dendritic cell, and Type 2 T helper cell. CAHM, DUXAP8, and RHPN1−AS1 were positively correlated with Activated CD4 T cells. These results indicated that the hypoxia-related lncRNA signature might be mainly expressed in the above immune cells.

Hypoxia has been reported to up-regulate the expression level of immune checkpoints such as *PDL1* to induce immune escape (Lequeux, et al., 2019). Hence, we also investigated the correlation between the hypoxia-related risk score and the expression levels of several critical immune checkpoints. Results showed that the mRNA expression levels of *PD1 (PDCD1)*, *PDL1 (CD274)*, *CTLA4*, *LAG3*, and *TIGIT* were consistently elevated in the hypoxia-related high-risk group in comparison with the low-risk counterpart (Supplementary Figures S13A–E). Meanwhile, the risk score was significantly positively correlated with the mRNA expression of *PD1(PDCD1)*, *PDL1(CD274)*, *CTLA4*, *LAG3*, and *TIGIT* (Figure 12B). the above evidence demonstrates that hypoxia indeed contributes to the tumor immune dysfunction and immune exclusion in HCC.

### Prediction of Immunotherapy Responsiveness and Targeted Drug Sensitivity

**FIGURE 13:**
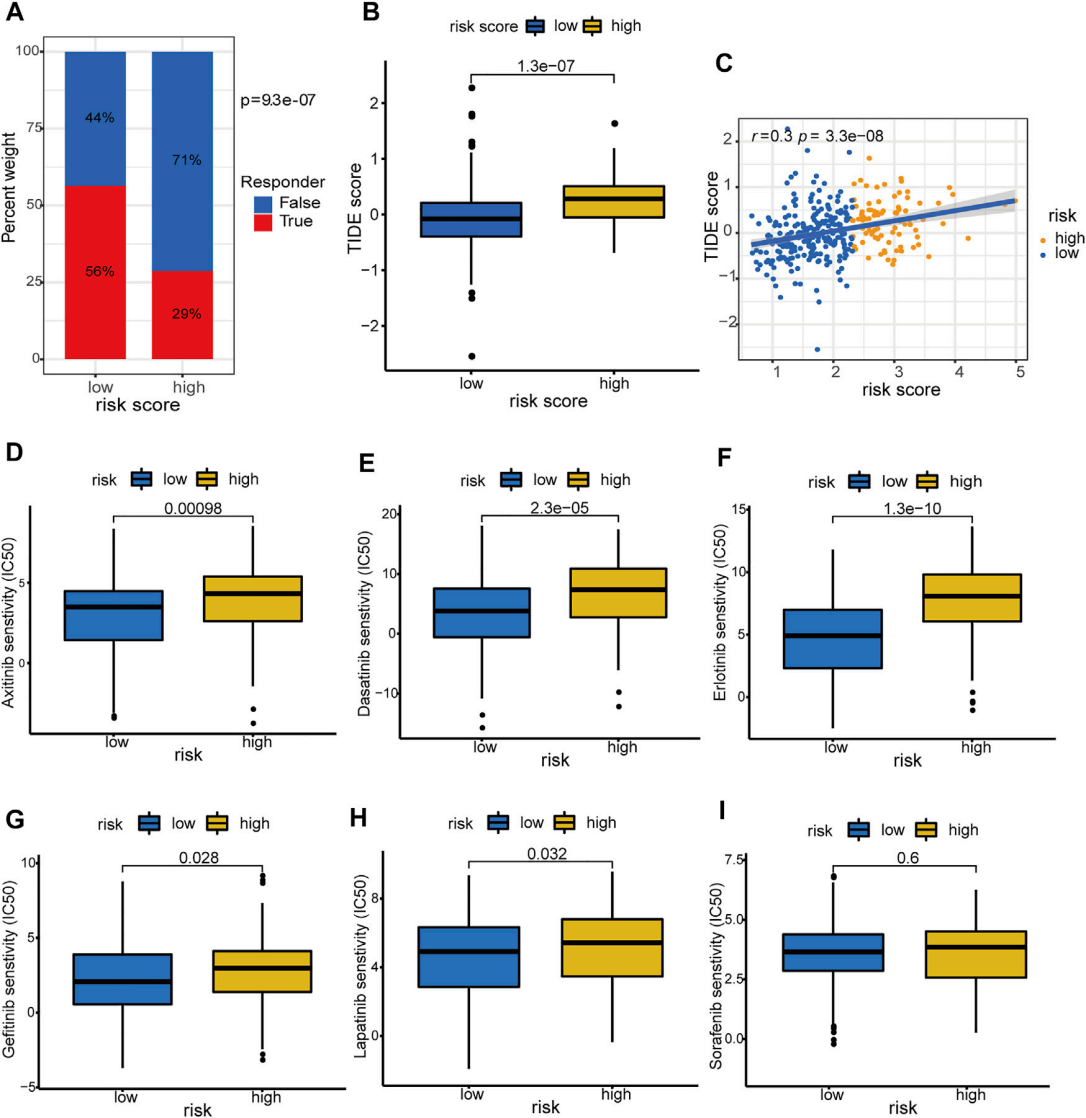
Prediction of immunotherapy response and targeted-drug sensitivity. **(A)** Comparison of predicted immunotherapeutic responder proportion and **(B)** TIDE score between the high- and low-risk groups. **(C)** Correlation between hypoxia-related risk score and TIDE score in TCGA-LIHC cohort. Comparisons of the IC_50_ values between the high- and low-risk groups for Axitinib **(D)**, Dasatinib **(E)**, Erlotinib **(F)**, Gefitinib **(G)**, Lapatinib **(H)**, and Sorafenib **(I)**, respectively. TIDE: Tumor Immune Dysfunction and Exclusion. IC_50_: half-maximal inhibitory concentration.

Accumulative evidence suggests that hypoxia can drive cancer cells to an immune resistance phenotype and is associated with resistance to immunotherapy (Abou Khouzam, et al., 2020). Hypoxia is also involved in the acquired chemoresistance during cancer chemotherapy (Akman, et al., 2021). Therefore, we investigated the association of the hypoxia-related lncRNA signature with immunotherapy response and targeted drug sensitivity in HCC. The low-risk group was predicted to hold a higher proportion of immunotherapeutic responders compared with the high-risk counterpart (56 versus 29%, chi-square test *p* = 9.3 e−07, Figure 13A; Supplementary Table S13). Patients with low-risk scores had lower TIDE scores, which means more responsive to the immunotherapy, compared with those with high-risk scores (*p* = 1.3 e−07, Figure 13B). Moreover, the hypoxia-related risk score has a significant positive correlation (r = 0.3 and *p* = 3.3 e−08) with the TIDE score (Figure 13C). These results demonstrated that the hypoxia-related lncRNA signature could distinguish the immunotherapeutic responders in HCC and had the potential to serve as a predictor of the immunotherapy response in patients with HCC. The drug sensitivity analyses revealed that patients in the low-risk group exhibited a significantly lower IC_50_ value of the several drugs including axitinib, dasatinib, erlotinib, gefitinib, and lapatinib (except for sorafenib) in contrast with the high-risk group (Figures 13D–I), suggesting a potential treatment sensitivity of these patients towards above drugs. According to these results, we conclude that the HRDELs-derived signature has the potential predictive ability of immunotherapy response and targeted drug sensitivity.

## Discussion

HCC accounts for approximately 90% of liver malignancies and possesses high mortality (Forner, et al., 2018). It is urgent to explore new prognostic biomarkers and potential therapeutic predictors of immunotherapeutic response for HCC. Studies have demonstrated that the hypoxic tumor microenvironment promotes tumor progression, metastasis, recurrence, and drug resistance (LaGory and Giaccia, 2016; Rankin and Giaccia, 2016). Another study (Zhang, et al., 2020) established a hypoxia-related gene signature connected with unfavorable prognosis and elevated recurrence rate in HCC. However, there is still a lack of hypoxia-related lncRNAs prognostic signature in HCC. lncRNAs play a crucial role in the hypoxia-response process of cancer cells (Choudhry, et al., 2016; Huan, et al., 2020), and the interplay between hypoxia and lncRNAs associates with tumor growth and metastasis (Wang, et al., 2021). Thus, we for the first time microdissected the hypoxia-related lncRNA landscape in HCC and identified three hypoxia-specific clusters which are strongly related to OS and DFS outcomes. We further established a robust and reliable hypoxia-related lncRNA signature associated with a poor prognosis in HCC. Time-dependent ROC curves illustrate that the constructed model is superior to age, AJCC stage, tumor pathological grade, and HIF-1A mRNA expression in the prognostic prediction of HCC. More importantly, we constructed a clinical nomogram including the HRDELs-derived signature and AJCC stage, and the nomogram model showed good discrimination, calibration, and clinical net benefit. These results demonstrated that the hypoxia-related lncRNA signature can improve the prognosis prediction in HCC and has good clinical practicability.

The prognostic signature comprises five hypoxia-related lncRNAs, which are all associated with poor clinical outcomes in HCC and their expression levels are elevated in HCC tumor tissues. *DUXAP8* promotes the growth and proliferation of HCC cell lines by suppressing Krüppel-like factor 2 (KLF2) expression (Jiang, et al., 2019). (Gao, et al., 2020) revealed that *MKLN1-AS* promoted HCC progression by acting on miR-654-3p, and down-regulation of *MKLN1-AS* inhibits the aggressive phenotype of HCC cells. *RHPN1-AS1* enhances the proliferation and invasion process of HCC cells by targeting miR-7-5p (Song, et al., 2020). The above evidence is consistent with our results and confirms that *DUXAP8*, *MKLN1-AS*, and *RHPN1-AS1* are crucial oncogenic lncRNAs in HCC. Notably, *CAHM* and *LINC00869* have not been reported in HCC yet and their role in HCC is worth further study to explore novel treatment targets.

Subsequently, we analyzed the underlying molecular mechanism related to the hypoxia-related lncRNAs. Unsurprisingly, the high-risk group exhibited increased HIF-1A mRNA expression compared to the low counterpart. HIF-1α plays a key role in the regulation of tumor progression, metastasis, and recurrence under hypoxic conditions (LaGory and Giaccia, 2016; Rankin and Giaccia, 2016). Hence, the constructed signature indeed reflects the hypoxia exposure level of HCC tissues. The risk score is also positively correlated with both the RNAss and DNAss, indicating the crucial role of hypoxia in contributing to the enhanced tumor stemness in HCC (Cui, et al., 2017). In addition, GSEA displays that the high-risk cohort exhibits more enriched scores in the “WNT_BETA_CATENIN_SIGNALING”, “PI3K_AKT_MTOR_SIGNALING”, and “EPITHELIAL_MESENCHYMAL_TRANSITION” pathways than the low-risk cohort. Hypoxia has been reported to promote EMT in HCC to induce immunosuppression and facilitate tumor metastasis (Ye, et al., 2016). Thus, we speculate that hypoxia-related lncRNAs may exert their action through the above oncogenic pathways to regulate the progression of HCC.

Tumor immune infiltration pattern contributes greatly to the progression of HCC. The CD8^+^ T cell is critical for anti-tumor immunity in HCC and can directly induce the death of tumor cells (Wei, et al., 2016). More abundance of CD8^+^ T cells is correlated with less recurrence and a longer recurrence-free survival time in HCC (Gabrielson, et al., 2016). Tumor-infiltrating B cells can positively mediate the antigen presentation process to induce tumor killing (Wouters and Nelson, 2018). In our study, the low-risk group with a better prognosis displays more abundance in CD8^+^ T cells and activated B cells than the high-risk group, and thus possesses elevated anti-tumor immunity. In contrast, the high-risk group exhibits a high fraction of immature dendritic cells and activated dendritic cells, which may be due to the phenomenon that the chronic hypoxic microenvironment exerts a stimulatory action on the immunoregulatory functions of immature dendritic cells (Pierobon, et al., 2013). Therefore, we conclude that the hypoxia-related lncRNA signature is tightly connected with the tumor immune microenvironment in HCC. The hypoxic tumor microenvironment supports tumor stemness, metastasis, and tumor immune escape (Chouaib, et al., 2017; Samanta and Semenza, 2018), and also up-regulates critical immune checkpoints expression such as PD1/PDL1 (Lequeux, et al., 2019). We also uncovered that the high-risk cluster exhibited elevated expression levels of *PD1*, *PDL1*, *CTLA4*, *LAG3*, and *TIGIT* compared to the low-risk counterpart, supporting the contribution of hypoxia to the tumor immune escape in HCC.

Hypoxia has been considered to drive cancer cells to an immune resistance phenotype and is associated with resistance to immunotherapy (Abou Khouzam, et al., 2020; Wu, et al., 2019). We also investigated the association of our constructed signature with immunotherapy response using the TIDE algorithm, which can effectively predict the treatment responsiveness of immune checkpoint blockade (Jiang, et al., 2018). A higher TIDE score means more T cell dysfunction or more exclusion of T cell infiltration and thus less response to immunotherapy. Notably, the low-risk group possesses more potential immunotherapeutic responders compared to the high-risk counterpart. We speculate that the low-risk group represents less hypoxic exposure and therefore is more responsive to immunotherapy. Additionally, the low-risk group exhibits a lower inhibitory concentration (IC_50_) value of Axitinib, Dasatinib, Erlotinib, Gefitinib, and Lapatinib, suggesting a higher sensitivity to these drugs than the high-risk group. Hypoxia aberrantly activates the HIF-1α pathway and several specific oncogenic pathways, inducing chemoresistance in cancer chemotherapy (Akman, et al., 2021; Kim and Lee, 2017). In line with these studies, the high-risk group retains more enriched scores in the “WNT_BETA_CATENIN_SIGNALING” and “PI3K_AKT_MTOR_SIGNALING” pathways, demonstrating the potential chemoresistance mechanism under the hypoxia condition in HCC. However, the IC_50_ value of sorafenib shows no statistical difference between the two groups. This phenomenon may be due to the intricate mechanism of sorafenib resistance including epigenetic modification, autophagy, ferroptosis, hypoxia, immune microenvironment (Tang, et al., 2020), and tumor genetic heterogeneity with HCC (Cabral, et al., 2020). Collectively, the hypoxia-related lncRNA signature has the potential to predict immunotherapy response and targeted drug sensitivity.

However, our present study has some limitations. Due to the absence of another public dataset of HCC patients with matched lncRNA expression profiles and complete survival data, the prognostic model was validated in an internal split testing dataset and lacked complete external validation. Thus, additional studies will be needed to further verify its reliable prognostic value. Meanwhile, the signature has been proved to possess the potential predictive capability of immunotherapy response by bioinformatical analysis, but well-designed clinical trials are required to further examine its performance. Additionally, *CAHM* and *LINC00869* are reported in HCC for the first time, their mechanism is worth further exploration by molecular function experiment.

In conclusion, the hypoxia-related lncRNA landscape correlates with clinical outcomes in patients with HCC. We established a reliable hypoxia-related lncRNAs signature that could accurately predict the clinical outcomes of HCC patients and correlate with immunotherapy response and targeted drug sensitivity, providing new insights for immunotherapy and targeted therapy in HCC.

## Data Availability

The original contributions presented in the study are included in the article/Supplementary Material, further inquiries can be directed to the corresponding author.

## Abbreviations

AUC, area under the curve; AJCC, American Joint Committee on Cancer; DFS, disease-free survival; DELs, differentially expressed lncRNAs; HCC, hepatocellular carcinoma; HRDELs, hypoxia-related differentially expressed lncRNAs; LASSO, Least absolute shrinkage and selection operator; LIHC, liver hepatocellular carcinoma; OS, overall survival; ROC, receiver operating characteristic; TCGA, The Cancer Genome Atlas; TIDE, The Tumor Immune Dysfunction and Exclusion.
