# Supplementary material for: Identifying a Hypoxia-Related Long Non-Coding RNAs Signature to Improve the Prediction of Prognosis and Immunotherapy Response in Hepatocellular Carcinoma
Source: Front Genet. 2021 Nov 30;12:785185. doi: 10.3389/fgene.2021.785185 (PMC8669612; doi:10.3389/fgene.2021.785185)
Supplement: Supplementary file 1 [file DataSheet1.PDF]

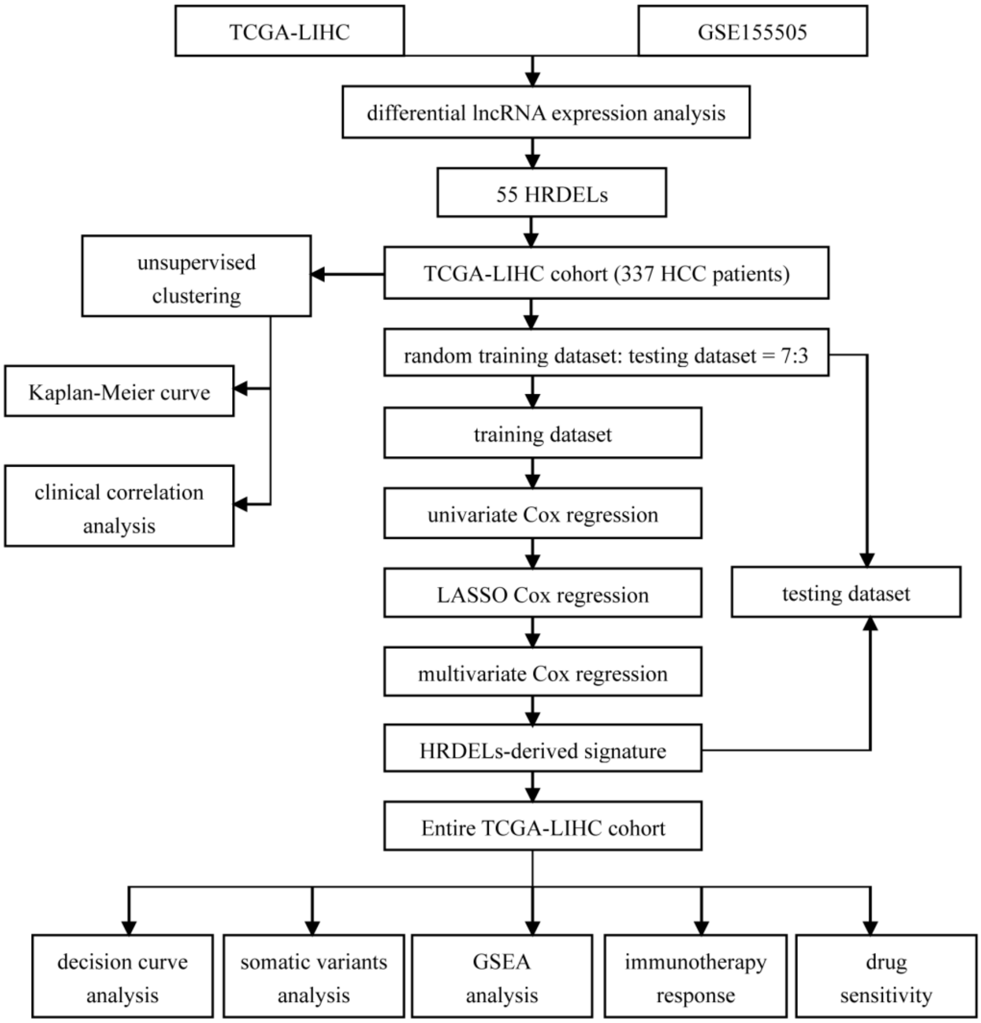


Figure S1. The flow chart of the present study. HRDELs: hypoxia-related differentially expressed lncRNAs. TCGA: The Cancer Genome Atlas. LIHC: liver hepatocellular carcinoma. HCC: hepatocellular carcinoma.

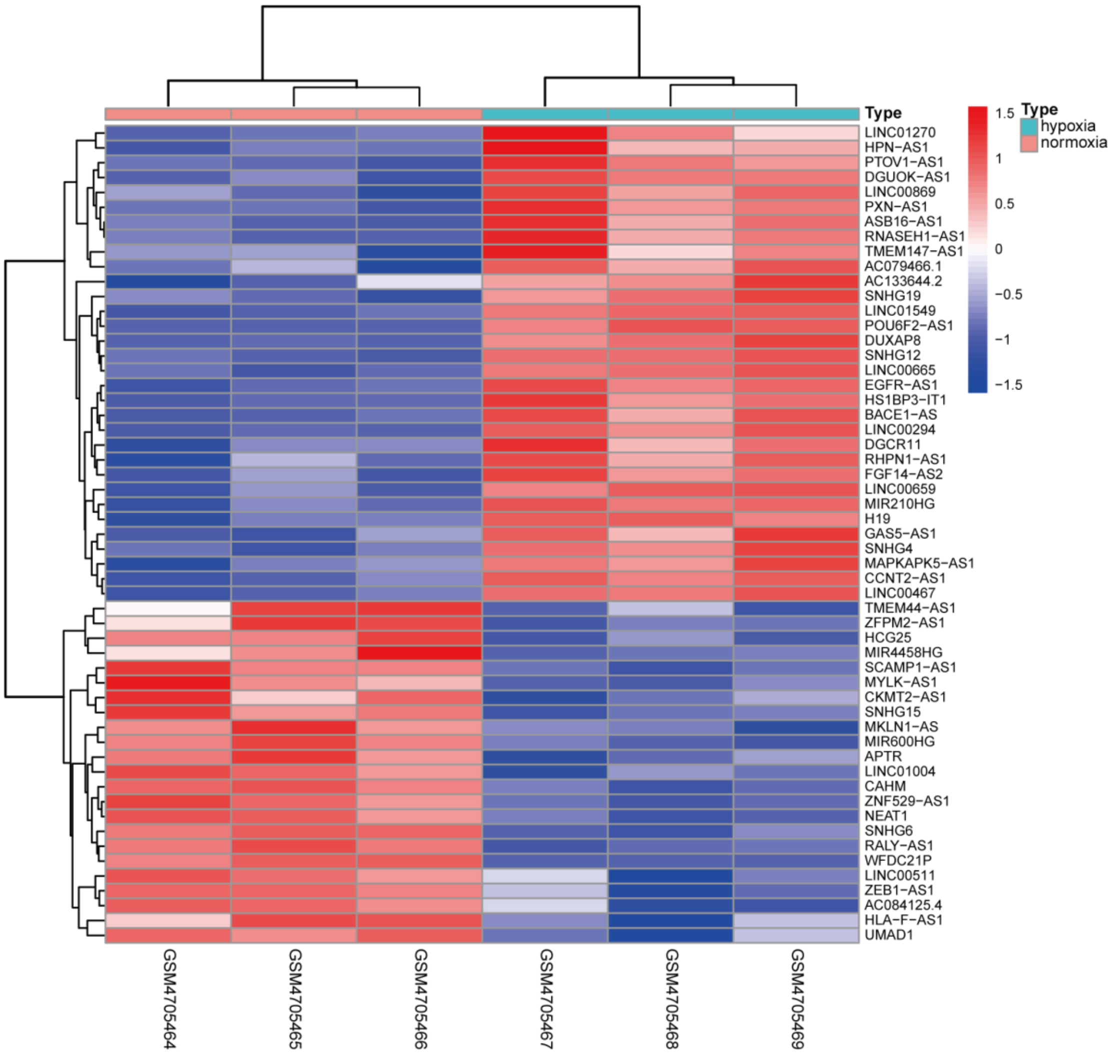


Figure S2. Heatmap of the expression levels of 55 HRDELs between HCC cells treated with hypoxia or normoxia in GSE155505. HRDELs: hypoxia-related differentially expressed lncRNAs. HCC: hepatocellular carcinoma.

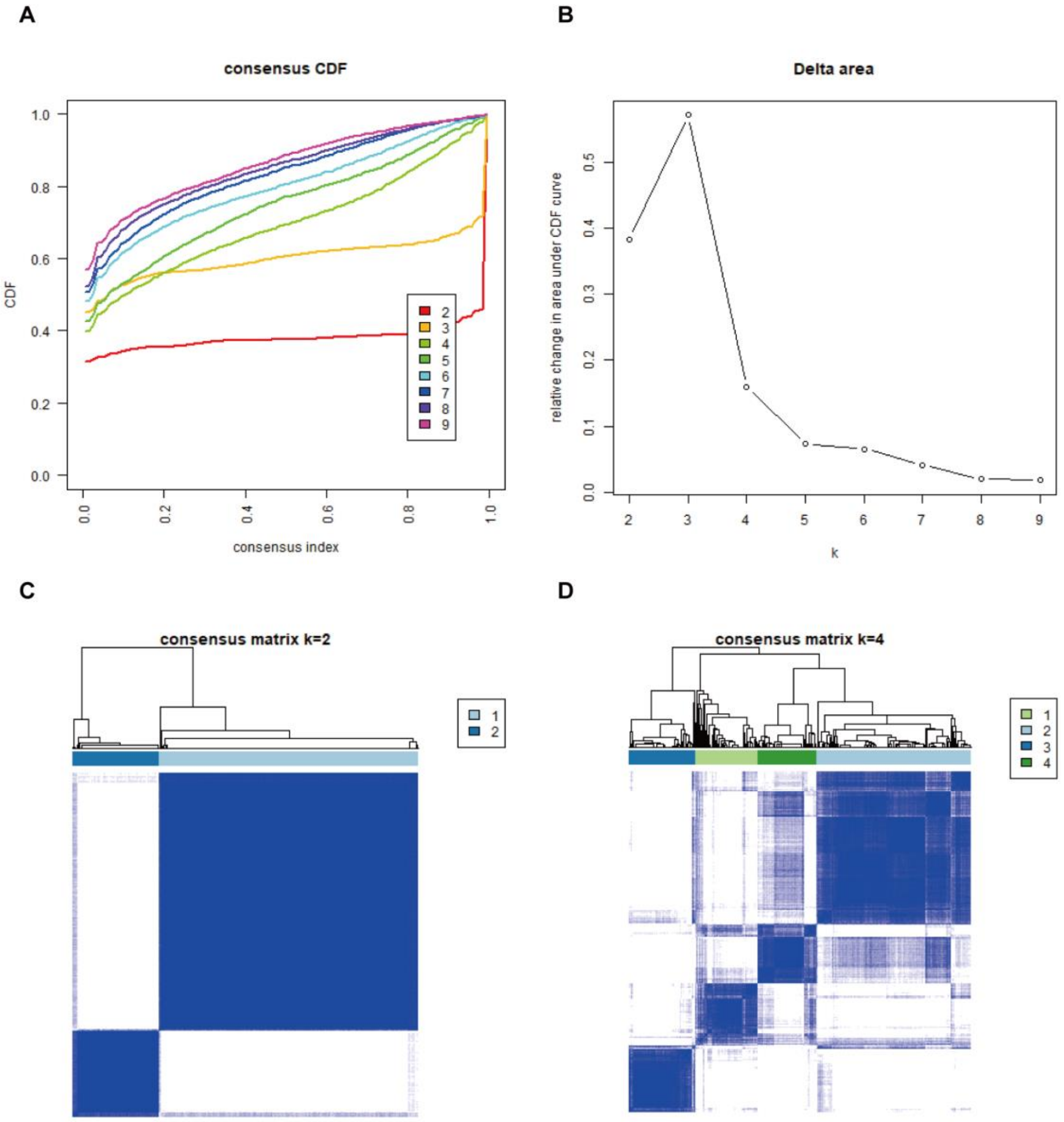


Figure S3. Microdissection of the hypoxia-related lncRNA landscape in the TCGA-LIHC cohort. **(A)** Consensus CDF curve and **(B)** Delta area curve of unsupervised clustering via the "K-means" method, when k = 3 representing the optimal cluster number. Consensus matrix plots of the expression levels of 55 HRDELs in patients with HCC, when k = 2 **(C)** and k = 4 **(D)**, respectively. CDF: Cumulative Distribution Function. HRDELs: hypoxia-related differentially expressed lncRNAs. HCC: hepatocellular carcinoma.

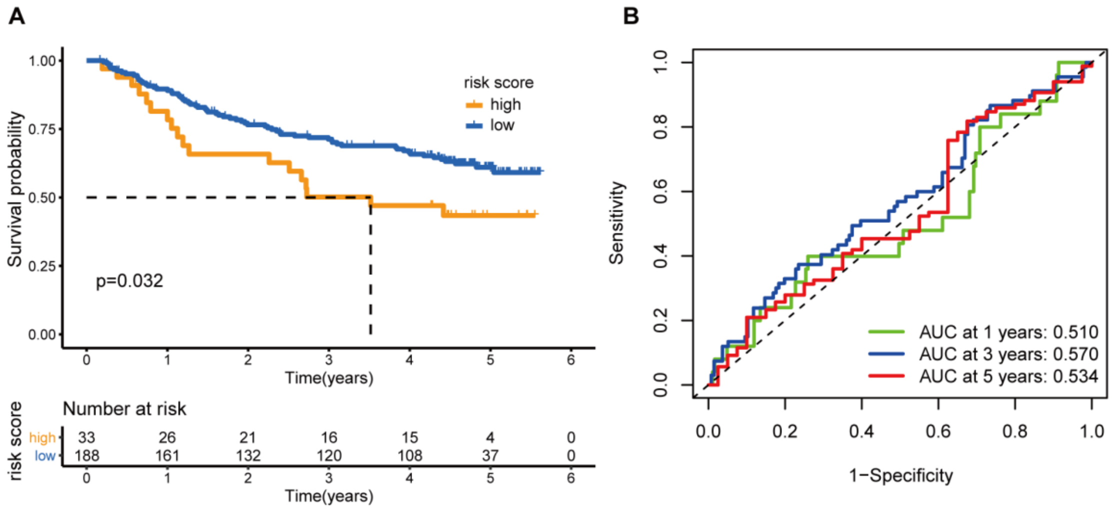


Figure S4. External validation of the hypoxia-related lncRNA signature in GSE14520-GPL3921. **(A)** Kaplan-Meier curves and log-rank test p-value. **(B)** The AUCs of the time-dependent ROC curves.

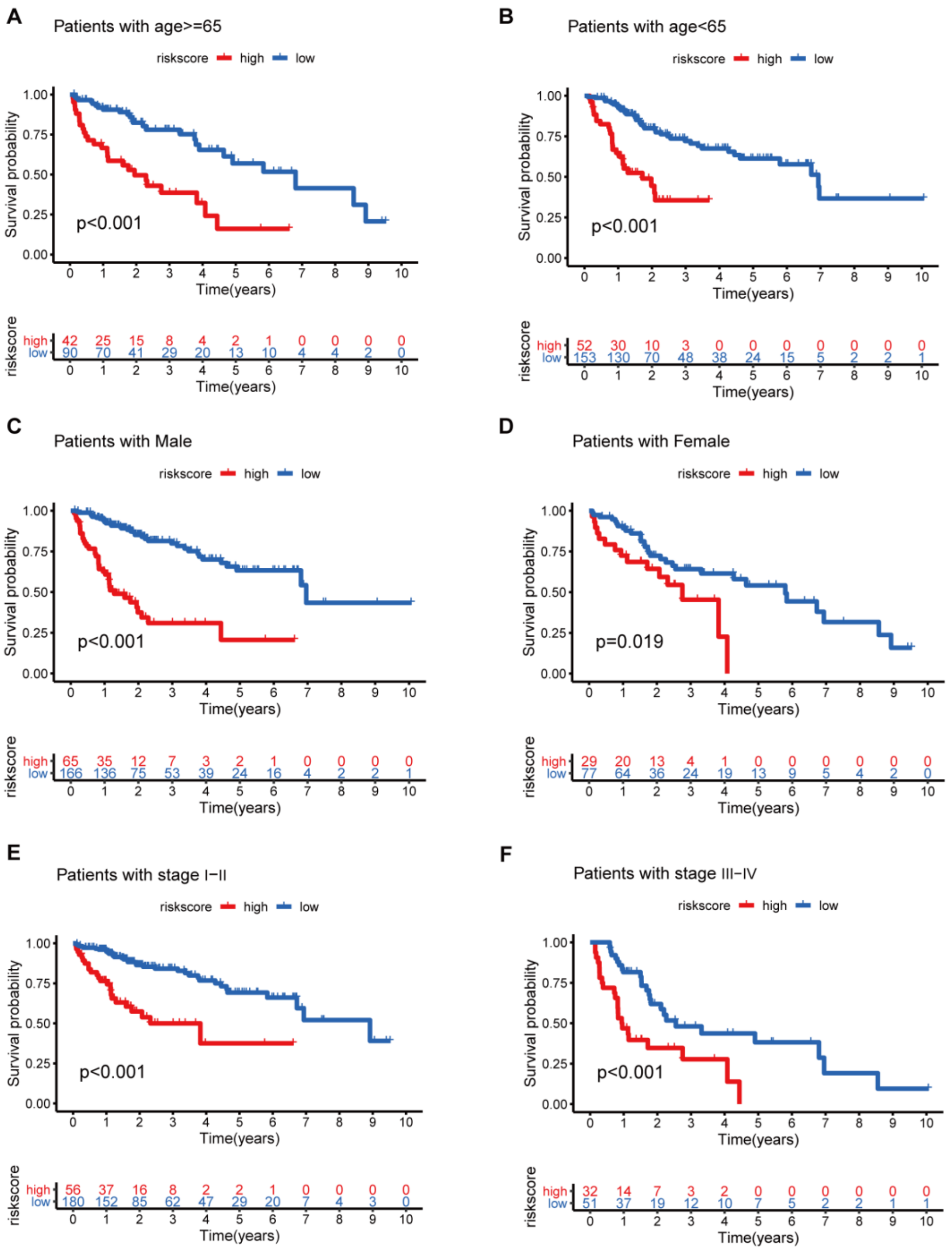


Figure S5. Survival analyses for the hypoxia-related lncRNA signature in different clinical subgroups of age (**A-B**), sex (**C-D**), and AJCC stage (**E-F**) in the entire TCGA-LIHC cohort. AJCC: American Joint Committee on Cancer.

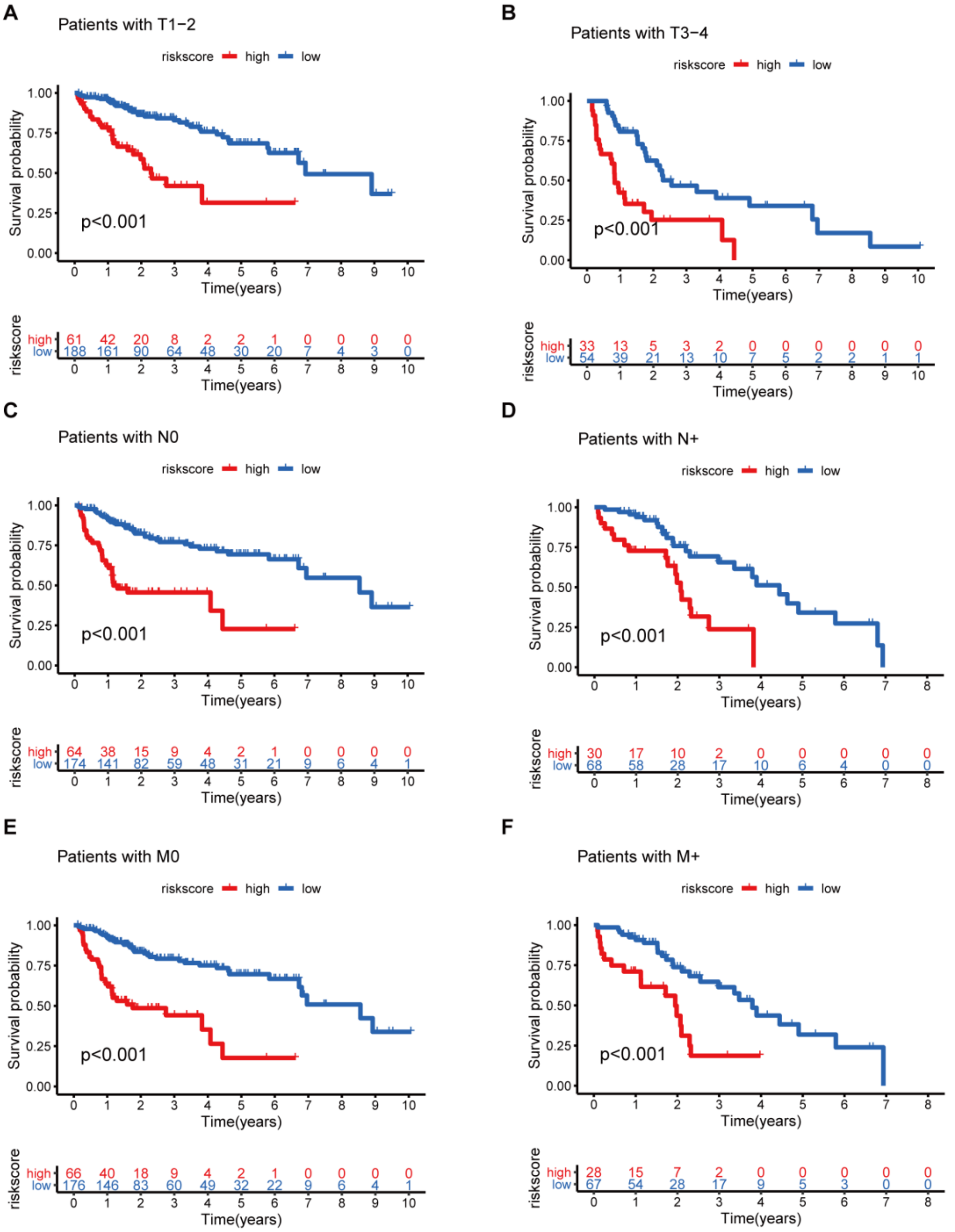


Figure S6. Survival analyses for the hypoxia-related lncRNA signature in different clinical subgroups of pathological T (**A-B**), N (**C-D**), and M (**E-F**) in the entire TCGA-LIHC cohort.

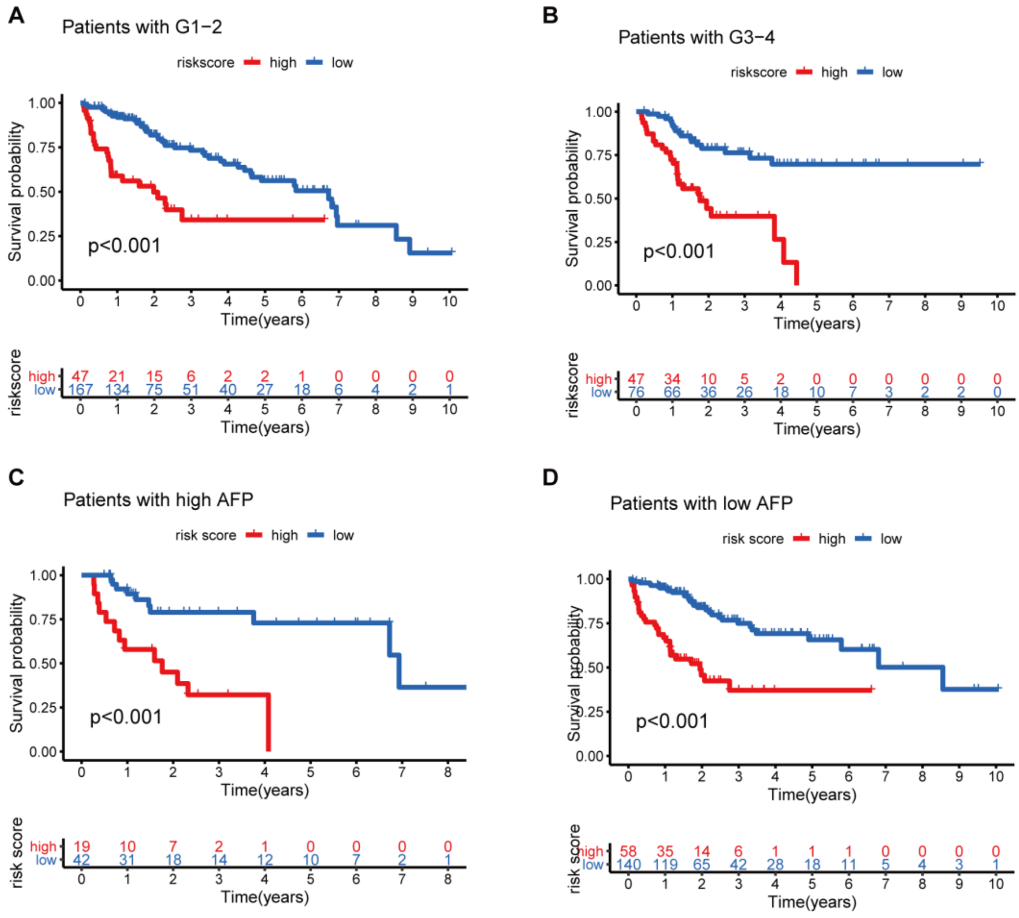


Figure S7. Survival analyses for the hypoxia-related lncRNA signature in different clinical subgroups of tumor histopathological grade (**A-B**) and AFP level (**C-D**) in the entire TCGA-LIHC cohort. AFP: Alpha-fetoprotein. G1-2: grade 1-2. G3-4: grade 3-4.

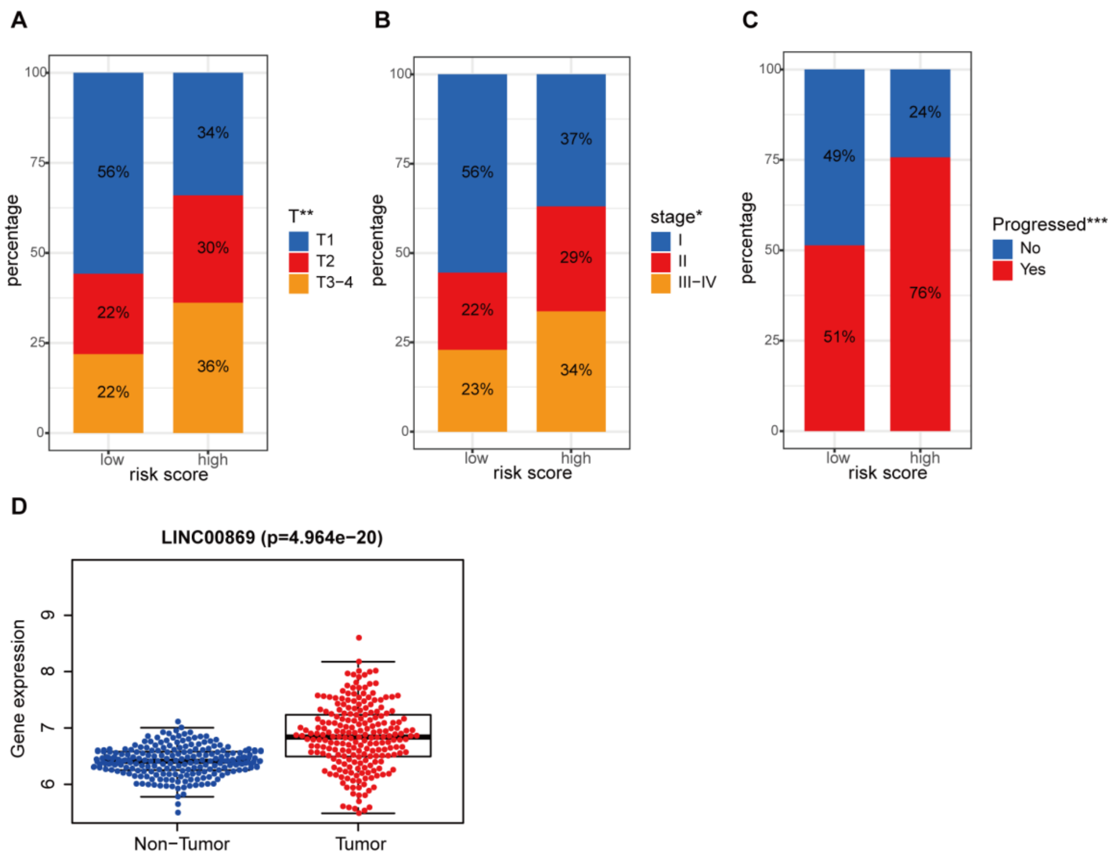


Figure S8. The distribution differences of the hypoxia-related risk groups among clinical parameters. Comparisons of the distribution differences of the hypoxia-related risk groups in pathological T (**A**), AJCC stage (**B**), and "Progressed (Ye/No)" (**C**). (**D**) Comparison of the expression level of LINC00869 between the HCC tumor samples and non-tumor samples in GSE14520-GPL3921. AJCC: American Joint Committee on Cancer. HCC: Hepatocellular carcinoma. ****, p<0.0001; ***, p<0.001; **, p<0.01; *, p<0.05.

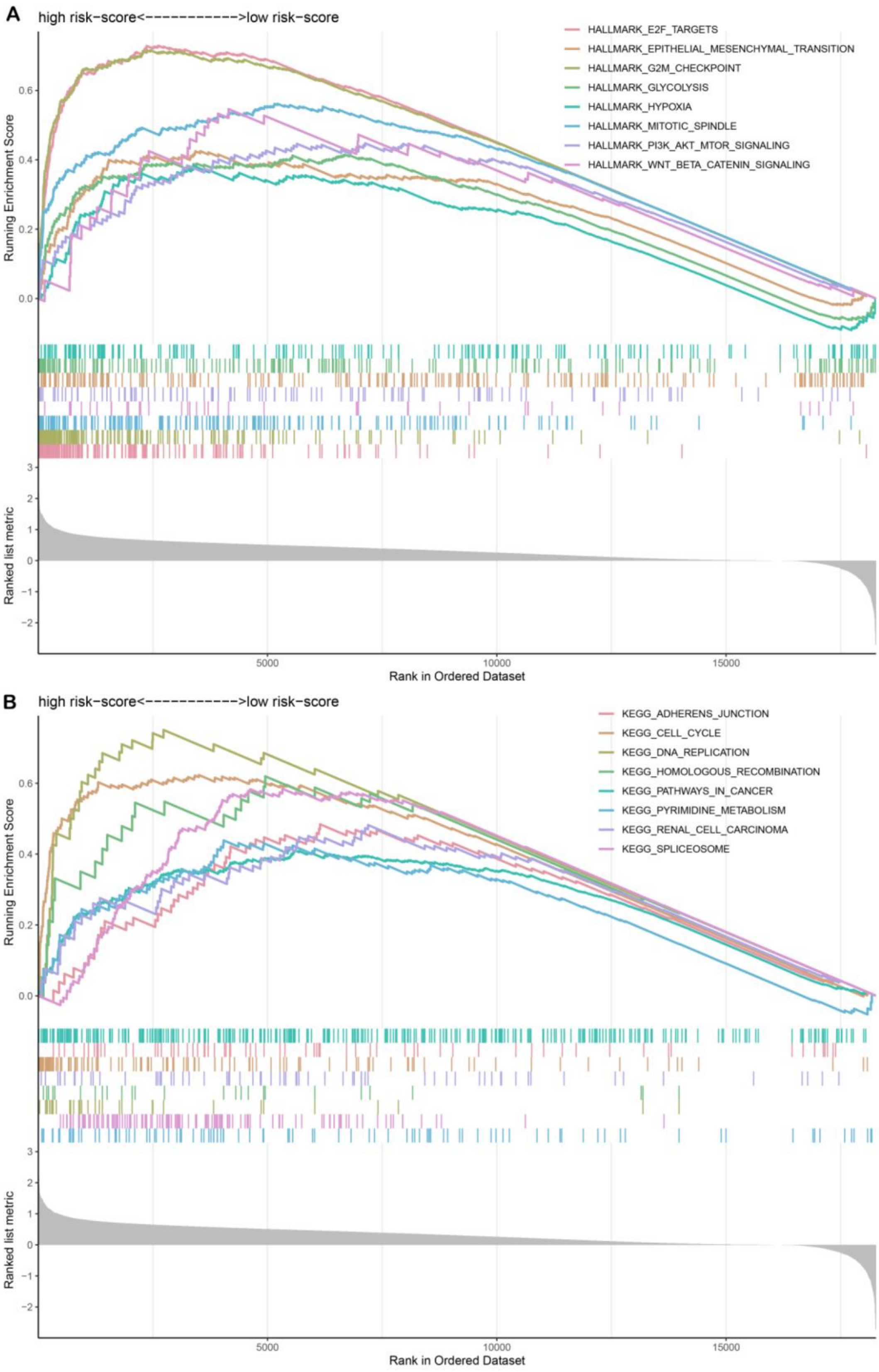


Figure S9. Identifying the differentially enriched pathways between the two risk groups using the GSEA method. Significantly enriched pathways of the hallmark gene sets **(A)** and KEGG pathway gene sets **(B)** in the hypoxia-related high-risk group compared with the low-risk group. GSEA: gene set enrichment analysis.

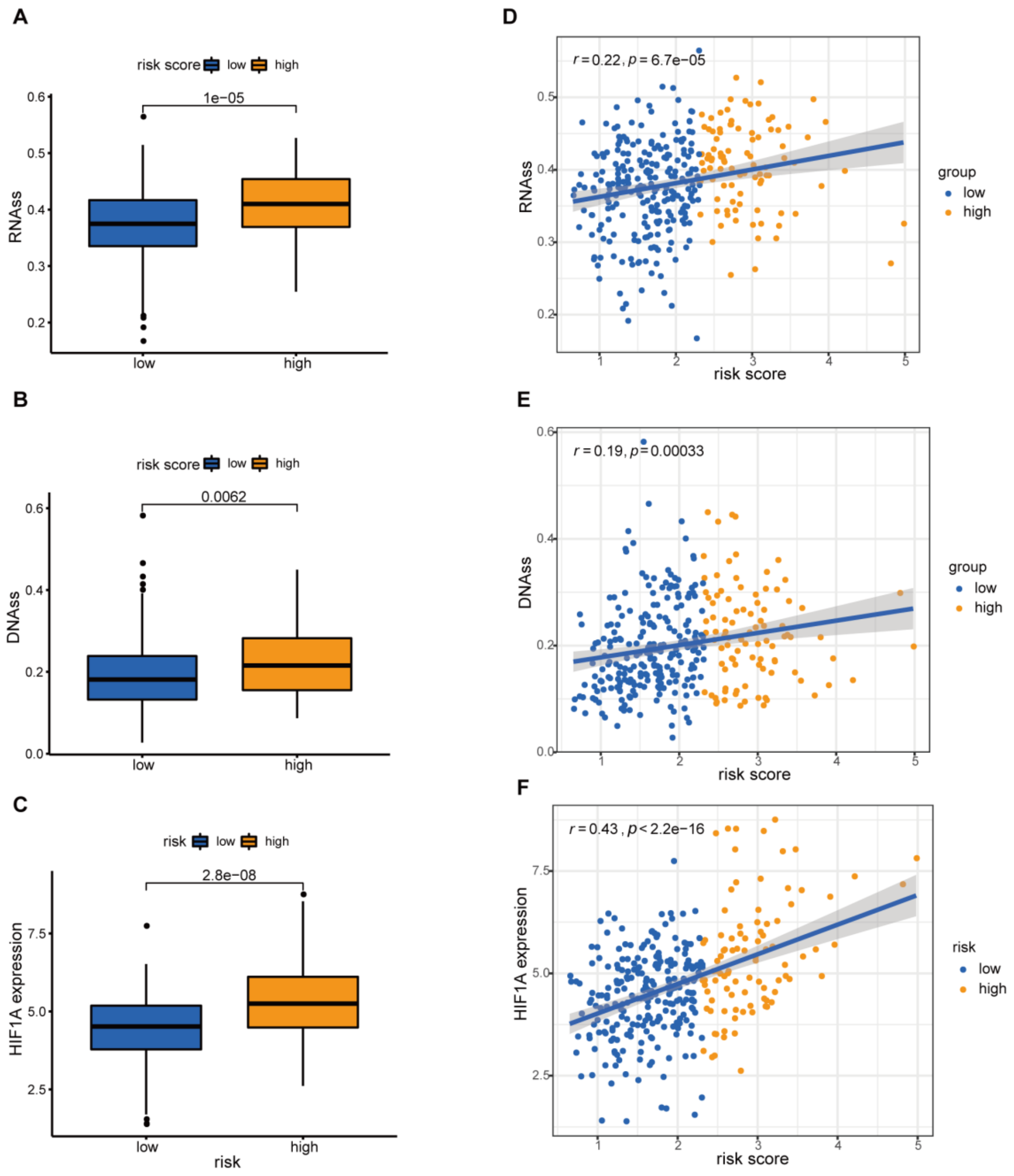


Figure S10. Association of the hypoxia-related lncRNA signature with RNAss, DNAss, and HIF1A mRNA expression level. Comparison of the RNAss **(A)**, DNAss **(B)**, and HIF1A mRNA expression **(C)** between the two groups. Correlation between the hypoxia-related risk score and RNAss **(D)**, DNAss **(E)**, and HIF1A mRNA expression **(F)**, respectively. RNAss: RNA-based stemness scores. DNAss: DNA methylation-based stemness scores.

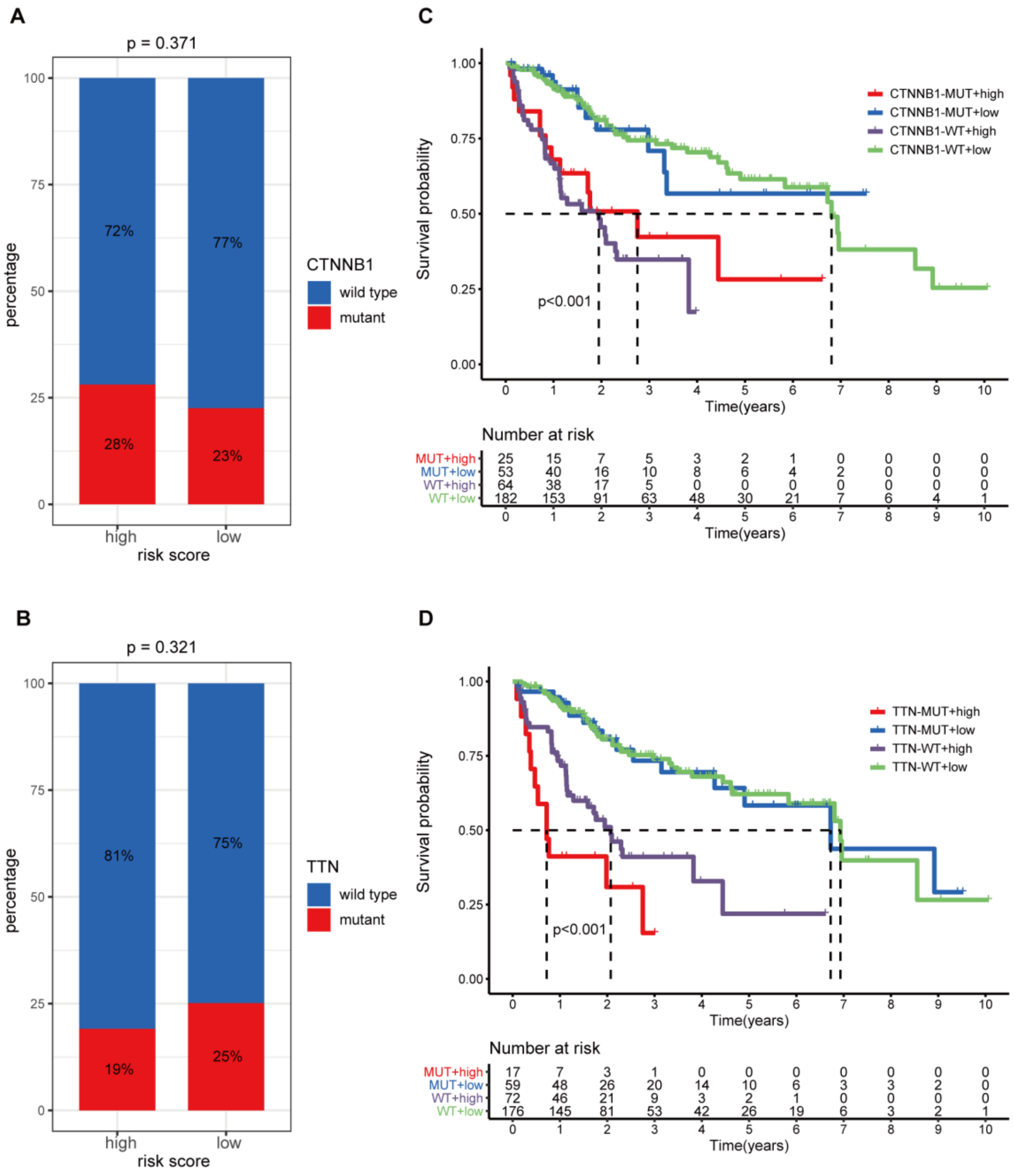


Figure S11. Somatic variants analyses of patients in TCGA-LIHC cohort. Comparisons of mutational frequency differences of CTNNB1**(A)** and TTN **(B)** between hypoxia-related high-risk and low-risk groups. **(C)** Survival analyses of the different clinical subgroups stratified by CTNNB1 status and hypoxia-related risk score. **(D)** Survival analyses of the different clinical subgroups stratified by TTN status and hypoxia-related risk score. MUT: mutant. WT: wild type. high: high-risk score. low: low-risk score.

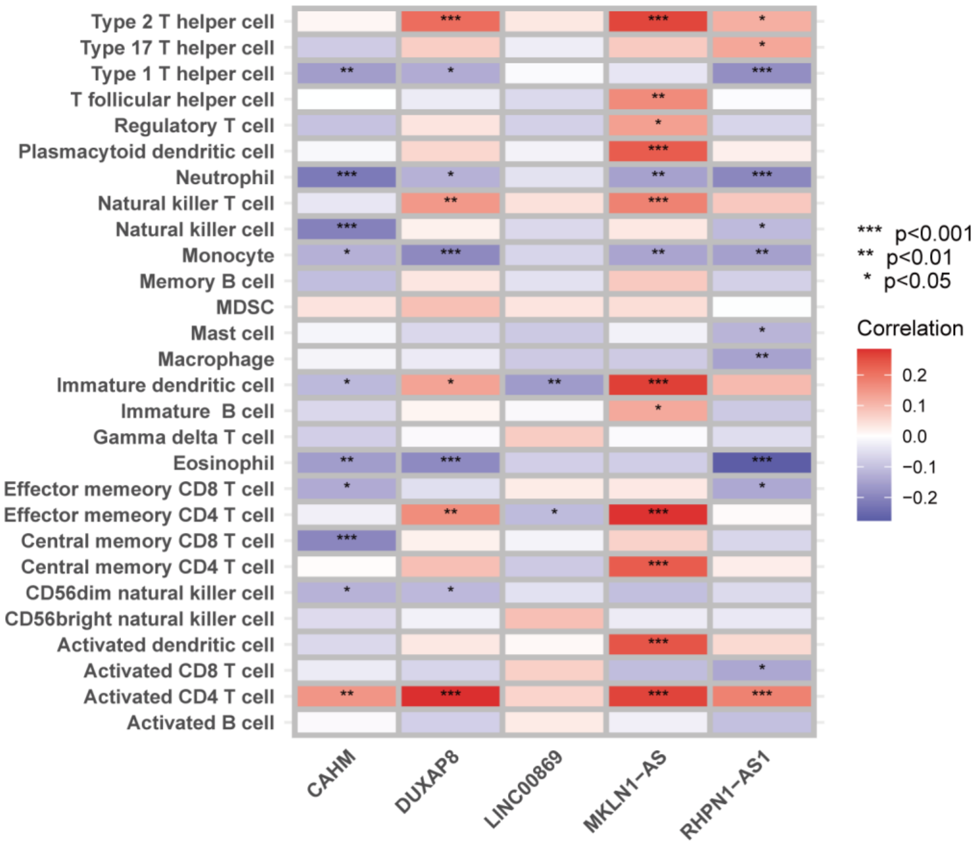


Figure S12. Heatmap of the correlations between the abundance of 28 immune cells and the expression levels of the five key lncRNAs in the hypoxia-related signature. ****, p<0.0001; ***, p<0.001; **, p<0.01; *, p<0.05.

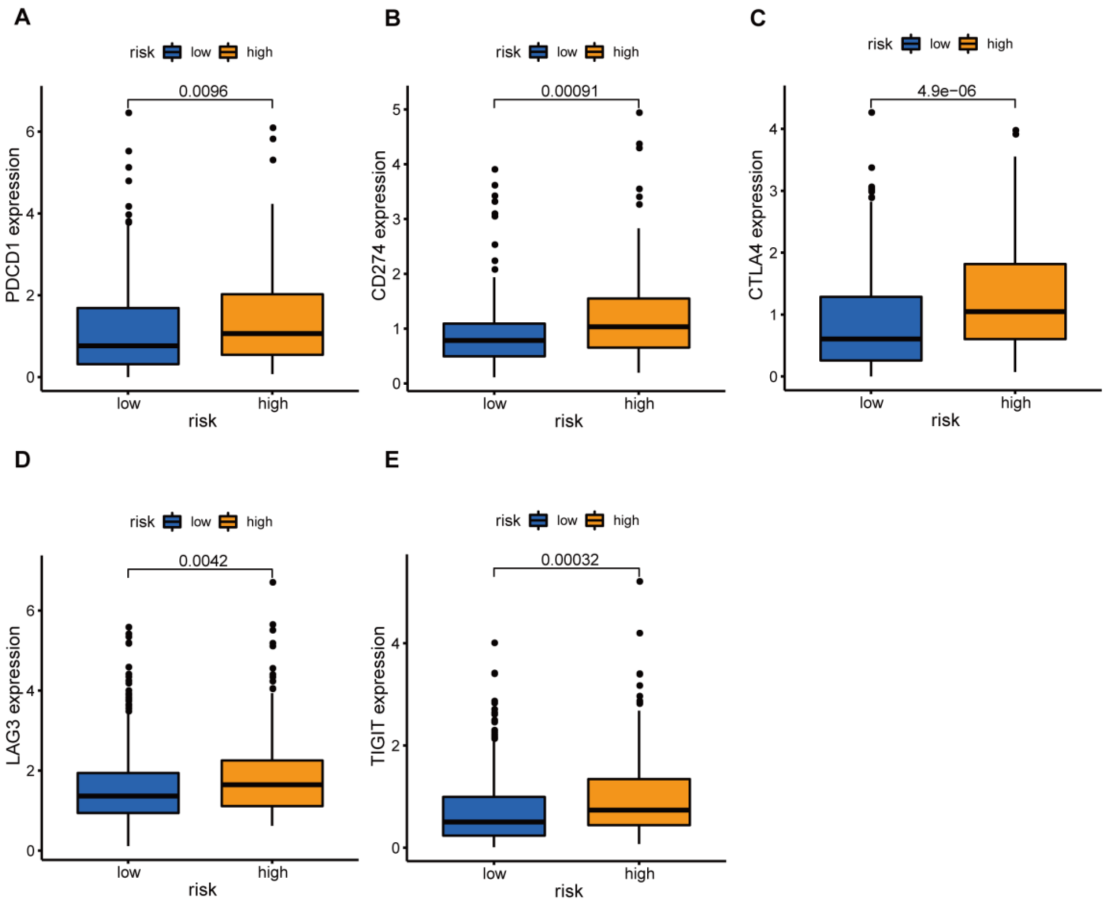


Figure S13. Comparisons of the mRNA expression levels of immune checkpoints. Comparisons of the mRNA expression levels of PDCD1 **(A)**, CD274 **(B)**, CTLA4 **(C)**, LAG3 **(D)**, and TIGIT **(E)** between the hypoxia-related risk-low and risk-high groups, respectively.
